# Lack of Replication of the *GRIN2A*-by-Coffee Interaction in Parkinson Disease

**DOI:** 10.1371/journal.pgen.1004788

**Published:** 2014-11-20

**Authors:** Ismaïl Ahmed, Pei-Chen Lee, Christina M. Lill, Susan Searles Nielsen, Fanny Artaud, Lisa G. Gallagher, Marie-Anne Loriot, Claire Mulot, Magali Nacfer, Tian Liu, Joanna M. Biernacka, Sebastian Armasu, Kari Anderson, Federico M. Farin, Christina Funch Lassen, Johnni Hansen, Jørgen H. Olsen, Lars Bertram, Demetrius M. Maraganore, Harvey Checkoway, Beate Ritz, Alexis Elbaz

**Affiliations:** 1 INSERM, Centre for research in Epidemiology and Population Health, U1018, Biostatistics team, Villejuif, France; 2 Univ Paris-Sud, UMRS 1018, Paris, Villejuif, France; 3 Department of Health Care Management, College of Healthcare Administration and Management, National Taipei University of Nursing Health Sciences, Taipei, Taiwan; 4 Department of Vertebrate Genomics, Neuropsychiatric Genetics Group, Max Planck Institute for Molecular Genetics, Berlin, Germany; 5 Department of Neurology, Focus Program Translational Neuroscience, University Medical Center of the Johannes Gutenberg University Mainz, Mainz, Germany; 6 Department of Environmental and Occupational Health Sciences, University of Washington, Seattle, Washington, United States of America; 7 INSERM, Centre for research in Epidemiology and Population Health, U1018, Social and occupational determinants of health, Villejuif, France; 8 Univ de Versailles St-Quentin, UMRS 1018, Versailles, France; 9 Assistance Publique Hôpitaux de Paris, Hôpital Européen Georges Pompidou, Service de Biochimie, Paris, France; 10 Université Paris Descartes, Inserm, UMR-S1147, Médecine personnalisée, Pharmacogénomique et optimisation thérapeutique, Paris, France; 11 Centre de ressources biologiques (CRB) Epigenetec, Paris, France; 12 Max Planck Institute for Human Development, Berlin, Germany; 13 Department of Health Sciences Research, Mayo Clinic, Rochester, Minnesota, United States of America; 14 Danish Cancer Society Research Center, Danish Cancer Society, Copenhagen, Denmark; 15 Department of Medicine, School of Public Health, Imperial College London, London, United Kingdom; 16 Department of Neurology, NorthShore University HealthSystem, Evanston, Illinois, United States of America; 17 Department of Family and Preventive Medicine, University of California, San Diego, La Jolla, California, United States of America; 18 Department of Epidemiology, Fielding School of Public Health, University of California Los Angeles, Los Angeles, California, United States of America; The Wellcome Trust Centre for Human Genetics, University of Oxford, United Kingdom

## Overview

The etiology of Parkinson disease (PD) involves both genetic susceptibility and environmental exposures. In particular, coffee consumption is inversely associated with PD but the mechanisms underlying this intriguing association are unknown. According to a recent genome-wide gene–environment interaction study, the inverse coffee–PD association was two times stronger among carriers of the T allele of SNP rs4998386 in gene *GRIN2A* than in homozygotes for the C allele. We attempted to replicate this result in a similarly sized pooled analysis of 2,289 cases and 2,809 controls from four independent studies (Denmark, France, Seattle-United States (US), and Rochester-US) with detailed caffeinated coffee consumption data and rs4998386 genotypes. Using a variety of definitions of coffee drinking and statistical modeling techniques, we failed to replicate this interaction. Notably, whereas in the original study there was an association between rs4998386 and coffee consumption among controls, but not among cases, none of the datasets analyzed here indicated an association between rs4998386 and coffee consumption among controls. Based on large, well-characterized datasets independent from the original study, our results are not in favor of an interaction between caffeinated coffee consumption and rs4998386 for PD risk and suggest that the original finding may have been driven by an association of coffee consumption with rs4998386 in controls. The next years will likely see an increasing number of papers examining gene–environment interactions at the genome-wide level, which poses important methodological challenges. Our findings underline the need for a careful assessment of the findings of such studies.

## Introduction

Genome-wide association studies (GWAS) have identified thousands of genetic risk variants for common diseases, which typically explain only a small proportion of the underlying heritability [1]. Unexplained or missing heritability could be partly due to gene–environment interactions. PD is a good example of a disease for which numerous susceptibility loci [2] and putative risk or protective environmental factors [3] have been identified and may interact. Among environmental factors, there is robust epidemiological evidence that coffee consumption is inversely associated with PD independently of smoking [4]. Caffeine is hypothesized to account for this association because it is an adenosine A_2A_-receptor antagonist, and this family of agents has been shown to be neuroprotective and attenuate loss of dopaminergic neurons in animal models of PD [5]; however, other explanations for this association, including reverse causation or confounding, cannot be discarded.

A recent genome-wide gene–environment interaction study in PD (testing 811,597 single nucleotide polymorphisms [SNPs] across 1,458 cases and 931 controls) used a joint test of marginal association and gene–environment interaction [6], followed by analyses stratified by coffee consumption, to identify modifiers of the coffee-PD association [7]. The inverse association between coffee and PD was about two times stronger among carriers of the rare T allele of rs4998386 in *GRIN2A* than in homozygotes for the major C allele (odds ratio (OR) for interaction, OR_interaction_ = 0.52, p = 4×10^−3^). This finding was replicated in a pooled analysis of three independent US datasets (1014 cases, 1917 controls; OR_interaction_ = 0.48, p = 5×10^−4^). The authors concluded that the inclusion of coffee consumption in their analyses to test for an interaction with rs4998386 allowed them to uncover one of the most important PD susceptibility genes, not previously identified in GWAS due to its small overall effect. *GRIN2A* encodes a subunit of the N-methyl-D-aspartate (NMDA) glutamate receptor and regulates excitatory neurotransmission in the brain. The authors considered it to be biologically plausible that *GRIN2A* plays a role in PD through an interaction with caffeinated coffee and suggested that *GRIN2A* genotypes may be a useful biomarker for pharmacogenetic studies on prevention and treatment in PD.

The study by Hamza et al. [7] represents one of the first published attempts to identify gene–environment interactions at a genome-wide scale, a challenging task given the requirement of very large sample sizes with exposure data [8]. The results from this study are of great interest as they may provide insight into the PD–coffee association and thus the underlying pathophysiology of PD. Analyses of gene–environment interactions can be performed through a variety of approaches [8], and, to better understand the findings presented by Hamza et al. [7], we performed a re-analysis of their data by examining the association between coffee and rs4998386 separately in cases and controls (Table S1). We found a strong positive association in controls between rs4998386-T and heavy coffee drinking (OR = 1.48, 95% CI = 1.23, 1.78, p = 3×10^−5^), thus suggesting that *GRIN2A*-rs4998386-T is associated with an increased likelihood of drinking coffee among persons free of PD. On the contrary, among PD cases, heavy coffee drinking tended to be less frequent in carriers of the rs4998386-T allele, but this association was not statistically significant (OR = 0.82, 95% CI = 0.65, 1.03, p = 0.08). Therefore, it appears that the interaction between rs4998386 and coffee consumption was in part explained by a positive association between the rs4998386-T allele and coffee consumption among controls, but not among PD cases.

Because of the well-described constraints of genome-wide gene–environment interaction analyses [8] and of this somewhat unusual pattern of gene–environment interaction, our objective was to replicate these findings by pooling data from four independent and well-characterized studies, three of them population-based, which had collected detailed coffee data.

## Results

Our analyses comprised 2,289 cases and 2,809 controls with complete data on coffee consumption, *GRIN2A*-rs4998386, and ever smoking. Rs4998386 genotypes were in Hardy-Weinberg equilibrium (HWE) in controls from each dataset (p≥0.05) and the frequency of the T allele was similar in controls across all studies (ranging from 8.7% to 11.8%). Rs4998386 was not associated with PD in any of the four datasets (Table S2). Danish participants had the highest level of coffee drinking. Ever coffee drinking was statistically significantly inversely associated with PD in the French and Danish datasets; in the Seattle-US dataset, PD cases were less frequently heavy coffee drinkers than controls, and there was no statistically significant association of coffee drinking and PD in the Rochester-US dataset (Table S2). Ever smoking was inversely associated with PD in all studies (Table S2). In pooled marginal association analyses of the French, Danish, and Seattle-US studies, rs4998386 showed no evidence for association with PD risk while ever coffee drinking was inversely associated with PD, showing a dose-response relation for all coffee variables (Table S3).

Table S4 shows the cross-tabulation of rs4998386 and coffee drinking by case-control status and dataset. Regardless of the definition of coffee drinking, there was no consistent significant departure from multiplicative effects of rs4998386 and coffee drinking in any of the individual datasets (Table 1, Table S5). In pooled analyses of the French, Danish, and Seattle-US datasets (Table 1), the inverse association with ever coffee drinking was stronger among CT+TT carriers (OR = 0.73/1.32 = 0.55) compared to CC carriers (OR = 0.77), but the difference was not statistically significant (OR_interaction_ = 0.72, p = 0.18). In analyses based on quantitative characteristics of coffee drinking, there was no evidence of statistically significant interactions, except for the category of 130–200 cupyears of coffee consumption (p = 0.038): the association with cupyears was stronger among CT+TT carriers (OR = 0.52/1.33 = 0.39) compared to CC carriers (OR = 0.70). Analyses based on the Rochester-US dataset and pooled analyses of all datasets revealed no statistically significant interactions. Analyses using the same approach to categorize coffee drinking as Hamza et al. [7] revealed no statistically significant interactions, except for participants from the Rochester-US dataset in the second quartile of cupyears; however, this result was only based on seven cases and 23 controls, this pattern was not apparent in the other studies, and there was no evidence of interaction at higher consumption levels (Table S6). In addition, interaction ORs with heavy coffee drinking tended to be greater than one, whereas Hamza et al. [7] reported interaction ORs smaller than one (Table S6). Pooled analyses of the French, Danish, and Seattle-US data using the empirical Bayes approach yielded results consistent with those of our main analyses; compared to the traditional case-control analysis, interaction ORs were generally closer to one and p-values greater (Table S7).

**Table 1:** Independent and joint effects of coffee drinking and *GRIN2A*-rs4998386 for Parkinson disease.

		France, Denmark, Seattle-US				Rochester-US				Pooled analysis			
				Interaction				Interaction				Interaction	
rs4998386	Coffee	OR (95% CI)^a^	p	OR (95% CI)^a^	p	OR (95% CI)^b^	p	OR (95% CI)^b^	p	OR (95% CI)^c^	p	OR (95% CI)^c^	p
	**Ever versus never**												
CC	Never	1.00 (Ref.)	-	-	-	1.00 (Ref.)	-	-	-	1.00 (Ref.)	-	-	-
CC	Ever	0.77 (0.62, 0.96)	0.017	-	-	1.03 (0.61, 1.73)	0.92	-	-	0.79 (0.65, 0.96)	0.016	-	-
CT, TT	Never	1.32 (0.84, 2.07)	0.23	1.00 (Ref.)	-	0.89 (0.31, 2.53)	0.83	1.00 (Ref.)	-	1.24 (0.82, 1.87)	0.30	1.00 (Ref.)	-
CT, TT	Ever	0.73 (0.57, 0.94)	0.014	0.72 (0.45, 1.16)	0.18	0.89 (0.41, 1.92)	0.77	0.97 (0.32, 2.99)	0.96	0.75 (0.60, 0.94)	0.015	0.77 (0.50, 1.19)	0.23
	**Cups per day**												
CC	Never	1.00 (Ref.)	-	-	-	1.00 (Ref.)		-	-	1.00 (Ref.)		-	-
CC	1 cup	0.98 (0.76, 1.26)	0.87	-	-	0.97 (0.55, 1.70)	0.91	-	-	0.95 (0.75, 1.19)	0.85	-	-
CC	2 cups	0.77 (0.61, 0.98)	0.034	-	-	1.14 (0.56, 2.32)	0.72	-	-	0.79 (0.63, 0.99)	0.094	-	-
CC	≥3 cups	0.64 (0.51, 0.81)	<0.001	-	-	1.12 (0.62, 2.04)	0.70	-	-	0.67 (0.54, 0.83)	0.38	-	-
CT, TT	Never	1.33 (0.85, 2.09)	0.22	1.00 (Ref.)	-	0.83 (0.29, 2.37)	0.73	1.00 (Ref.)	-	1.26 (0.84, 1.91)	0.32	1.00 (Ref.)	-
CT, TT	1 cup	0.99 (0.67, 1.47)	0.96	0.76 (0.42, 1.38)	0.37	0.64 (0.26, 1.57)	0.33	0.80 (0.23, 2.77)	0.72	0.85 (0.60, 1.21)	0.66	0.71 (0.42, 1.21)	0.21
CT, TT	2 cups	0.75 (0.53, 1.07)	0.11	0.73 (0.42, 1.28)	0.27	1.35 (0.47, 3.92)	0.58	1.43 (0.34, 6.12)	0.63	0.80 (0.58, 1.12)	0.26	0.81 (0.48, 1.35)	0.41
CT, TT	≥3 cups	0.59 (0.44, 0.79)	<0.001	0.69 (0.42, 1.14)	0.15	1.14 (0.43, 3.04)	0.79	1.22 (0.34, 4.35)	0.75	0.63 (0.48, 0.82)	0.19	0.74 (0.47, 1.17)	0.20
				Global test^d^	0.55			Global test^d^	0.76			Global test^d^	0.58
	**Cupyears**												
CC	Never	1.00 (Ref.)	-	-	-	1.00 (Ref.)		-	-	1.00 (Ref.)		-	-
CC	[0–65]	0.88 (0.69, 1.12)	0.30	-	-	0.92 (0.51, 1.64)	0.77	-	-	0.87 (0.69, 1.09)	0.22	-	-
CC	[65–130]	0.85 (0.67, 1.09)	0.20	-	-	1.19 (0.63, 2.25)	0.60	-	-	0.88 (0.70, 1.10)	0.27	-	-
CC	[130–200]	0.70 (0.55, 0.90)	0.006	-	-	1.06 (0.50, 2.22)	0.89	-	-	0.72 (0.57, 0.90)	0.005	-	-
CC	>200	0.61 (0.48, 0.79)	<0.001	-	-	1.21 (0.62, 2.37)	0.58	-	-	0.64 (0.51, 0.81)	0.0002	-	-
CT, TT	Never	1.33 (0.85, 2.08)	0.22	1.00 (Ref.)	-	0.90 (0.32, 2.58)	0.85	1.00 (Ref.)	-	1.26 (0.84, 1.91)	0.26	1.00 (Ref.)	-
CT, TT	[0–65]	0.98 (0.68, 1.42)	0.93	0.84 (0.48, 1.49)	0.56	0.72 (0.27, 1.90)	0.51	0.87 (0.24, 3.18)	0.83	0.91 (0.65, 1.28)	0.60	0.83 (0.50, 1.40)	0.49
CT, TT	[65–130]	0.72 (0.51, 1.03)	0.073	0.64 (0.37, 1.12)	0.12	0.78 (0.29, 2.11)	0.63	0.73 (0.18, 2.91)	0.65	0.72 (0.52, 1.00)	0.048	0.65 (0.39, 1.08)	0.092
CT, TT	[130–200]	0.52 (0.36, 0.74)	<0.001	0.55 (0.32, 0.97)	0.038	0.90 (0.30, 2.67)	0.84	0.94 (0.22, 3.98)	0.93	0.56 (0.40, 0.78)	0.0006	0.62 (0.37, 1.03)	0.064
CT, TT	>200	0.71 (0.51, 1.00)	0.049	0.88 (0.51, 1.50)	0.63	1.98 (0.62, 6.32)	0.25	1.81 (0.44, 7.47)	0.41	0.78 (0.56, 1.07)	0.12	0.95 (0.58, 1.57)	0.85
				Global test^d^	0.13			Global test^d^	0.75			Global test^d^	0.12
	**Years of coffee drinking**												
CC	Never	1.00 (Ref.)	-	-	-	1.00 (Ref.)		-	-	1.00 (Ref.)		-	-
CC	[0–37]	0.84 (0.65, 1.08)	0.17	-	-	1.00 (0.52, 1.90)	0.99	-	-	0.85 (0.68, 1.08)	0.18	-	-
CC	[37–45]	0.74 (0.58, 0.95)	0.018	-	-	1.27 (0.68, 2.38)	0.45	-	-	0.78 (0.62, 0.98)	0.034	-	-
CC	[45–53]	0.80 (0.63, 1.03)	0.084	-	-	0.85 (0.42, 1.73)	0.66	-	-	0.79 (0.63, 1.00)	0.051	-	-
CC	>53	0.71 (0.55, 0.92)	0.011	-	-	0.99 (0.46, 2.15)	0.98	-	-	0.73 (0.57, 0.92)	0.0089	-	-
CT, TT	Never	1.32 (0.84, 2.06)	0.23	1.00 (Ref.)	-	0.94 (0.33, 2.69)	0.90	1.00 (Ref.)	-	1.24 (0.82, 1.87)	0.30	1.00 (Ref.)	-
CT, TT	[0–37]	0.76 (0.53, 1.08)	0.12	0.69 (0.40, 1.18)	0.18	1.13 (0.35, 3.62)	0.83	1.21 (0.29, 5.00)	0.79	0.80 (0.58, 1.11)	0.19	0.76 (0.46, 1.25)	0.27
CT, TT	[37–45]	0.75 (0.53, 1.05)	0.093	0.77 (0.45, 1.32)	0.34	0.60 (0.21, 1.71)	0.34	0.50 (0.13, 2.01)	0.33	0.73 (0.53, 1.00)	0.052	0.75 (0.46, 1.25)	0.27
CT, TT	[45–53]	0.66 (0.46, 0.95)	0.025	0.62 (0.36, 1.09)	0.10	0.53 (0.15, 1.90)	0.33	0.67 (0.14, 3.26)	0.62	0.65 (0.46, 0.92)	0.014	0.65 (0.39, 1.11)	0.11
CT, TT	>53	0.79 (0.55, 1.14)	0.21	0.84 (0.48, 1.48)	0.54	1.38 (0.48, 3.99)	0.55	1.48 (0.38, 5.76)	0.57	0.84 (0.59, 1.18)	0.31	0.93 (0.55, 1.56)	0.78
				Global test^d^	0.48			Global test^d^	0.42			Global test^d^	0.44

a Odds ratios (OR) and 95% confidence intervals (CI) computed using unconditional logistic regression and adjusted for sex, age in quartiles, ever cigarette smoking, and dataset (1974 cases, 2494 controls).

b Odds ratios (OR) and 95% confidence intervals (CI) computed using conditional logistic regression and adjusted for sex, age in quartiles, and ever cigarette smoking (315 cases, 315 controls).

c Odds ratios (OR) and 95% confidence intervals (CI) computed by pooling individual data from the matched and unmatched case-control analyses and adjusted for sex, age in quartiles, ever cigarette smoking, and dataset (2289 cases, 2809 controls).

d Global test of interaction: p-values were computed using a likelihood ratio test that compared the likelihood of models with and without interaction terms.

We found similar results in sensitivity analyses when excluding TT homozygotes, adjusting for packyears of smoking (2140 cases, 2602 controls) or Mini-Mental State Examination (MMSE) (686 cases, 1,100 controls), or upon stratification by sex, median disease duration (<5 versus ≥5 years), and median age (≤70 versus >70 years) (data not shown). In addition, in the Seattle-US dataset, there was no interaction between rs4998386 and total caffeine intake from seven food and beverage sources.

Case-only analyses of the association between rs4998386 CT-TT genotypes and coffee consumption showed no evidence of association regardless of the coffee definition (Table 2). Table 3 shows the same set of analyses in controls. While there was no statistically significant association between rs4998386 and coffee, OR estimates tended to be greater than one.

**Table 2:** Association of coffee drinking with the CT-TT genotype of rs4998386 in the *GRIN2A* gene among Parkinson disease cases.

	France, Denmark, Seattle-US		Rochester-US		Pooled analysis	
Coffee	OR (95% CI)	p	OR (95% CI)	p	OR (95% CI)	p
**Never**	1.00 (Ref.)	-	1.00 (Ref.)	-	1.00 (Ref.)	-
**Ever**	1.05 (0.74, 1.48)	0.78	1.49 (0.71, 3.12)	0.30	1.12 (0.82, 1.53)	0.46
**Cups per day**						
Never	1.00 (Ref.)	-	1.00 (Ref.)	-	1.00 (Ref.)	-
1 cup	1.05 (0.69, 1.58)	0.83	1.10 (0.47, 2.57)	0.83	1.05 (0.73, 1.52)	0.79
2 cups	0.99 (0.67, 1.47)	0.95	2.27 (0.94, 5.52)	0.070	1.13 (0.79, 1.61)	0.52
≥3 cups	1.11 (0.76, 1.62)	0.59	1.51 (0.65, 3.53)	0.34	1.19 (0.84, 1.67)	0.33
	Global test	0.88	Global test	0.21	Global test	0.76
**Cupyears**						
Never	1.00 (Ref.)	-	1.00 (Ref.)	-	1.00 (Ref.)	-
[0–65]	1.08 (0.72, 1.61)	0.71	1.28 (0.53, 3.10)	0.59	1.12 (0.78, 1.61)	0.53
[65–130]	0.95 (0.63, 1.42)	0.79	1.19 (0.49, 2.91)	0.71	1.00 (0.69, 1.43)	0.98
[130–200]	0.98 (0.64, 1.48)	0.90	2.33 (0.90, 6.03)	0.08	1.11 (0.76, 2.62)	0.59
>200	1.27 (0.85, 1.92)	0.25	1.77 (0.70, 4.49)	0.23	1.36 (0.94, 1.97)	0.11
	Global test	0.43	Global test	0.41	Global test	0.32
**Years of coffee drinking**						
Never	1.00 (Ref.)	-	1.00 (Ref.)	-	1.00 (Ref.)	-
[0–37]	0.92 (0.61,1.39)	0.69	1.71 (0.67, 4.34)	0.26	1.02 (0.70, 1.48)	0.93
[37–45]	1.22 (0.81,1.82)	0.34	0.12 (0.46, 2.74)	0.81	1.22 (0.85, 1.75)	0.29
[45–53]	0.91 (0.60,1.39)	0.67	0.92 (0.30, 2.79)	0.88	0.94 (0.64, 1.38)	0.75
>53	1.17 (0.76,1.80)	0.47	2.24 (0.82, 6.08)	0.11	1.32 (0.89, 1.94)	0.16
	Global test	0.32	Global test	0.31	Global test	0.25

Odds ratios (OR) and 95% confidence intervals (CI) were computed using logistic regression and adjusted for sex, age in quartiles, ever cigarette smoking, and dataset. ORs compare the odds of carrying the rs4998386-CT or TT genotypes (outcome) in coffee drinkers (exposure) to the odds of carrying the rs4998386-CT/TT genotypes in never coffee drinkers among cases (France, Denmark, Seattle-US, N = 1974; Rochester, N = 315; pooled analysis, N = 2289).

**Table 3:** Association of coffee drinking with the CT-TT genotype of rs4998386 in the *GRIN2A* gene among controls.

	France, Denmark, Seattle-US		Rochester-US		Pooled analysis	
Coffee	OR (95% CI)	p	OR (95% CI)	p	OR (95% CI)	p
**Never**	1.00 (Ref.)	-	1.00 (Ref.)	-	1.00 (Ref.)	-
**Ever**	1.15 (0.81, 1.64)	0.45	1.46 (0.66, 3.21)	0.35	1.17 (0.85, 1.61)	0.34
**Cups per day**						
Never	1.00 (Ref.)	-	1.00 (Ref.)	-	1.00 (Ref.)	-
1 cup	1.19 (0.79, 1.81)	0.40	1.49 (0.64, 3.47)	0.35	1.23 (0.85, 1.77)	0.28
2 cups	1.08 (0.73, 1.60)	0.69	1.61 (0.60, 4.32)	0.35	1.11 (0.77, 1.59)	0.57
≥3 cups	1.18 (0.80, 1.73)	0.41	1.33 (0.55, 3.26)	0.53	1.18 (0.83, 1.68)	0.36
	Global test	0.78	Global test	0.77	Global test	0.71
**Cupyears**						
Never	1.00 (Ref.)	-	1.00 (Ref.)	-	1.00 (Ref.)	-
[0–65]	1.08 (0.72, 1.62)	0.71	1.31 (0.54, 3.18)	0.55	1.10 (0.77, 1.59)	0.60
[65–130]	1.11 (0.75, 1.67)	0.60	1.56 (0.63, 3.85)	0.34	1.15 (0.80, 1.66)	0.45
[130–200]	1.33 (0.90, 1.97)	0.15	1.98 (0.75, 5.24)	0.17	1.37 (0.96, 1.97)	0.084
>200	1.03 (0.68, 1.55)	0.89	1.11 (0.39, 3.18)	0.84	1.04 (0.71, 1.51)	0.85
	Global test	0.37	Global test	0.63	Global test	0.23
**Years of coffee drinking**						
Never	1.00 (Ref.)	-	1.00 (Ref.)	-	1.00 (Ref.)	-
[0–37]	1.13 (0.74, 1.72)	0.57	1.05 (0.37, 2.96)	0.93	1.12 (0.77, 1.65)	0.55
[37–45]	1.30 (0.87, 1.94)	0.20	1.95 (0.80, 4.75)	0.14	1.36 (0.94, 1.95)	0.10
[45–53]	1.14 (0.76, 1.70)	0.54	1.44 (0.51, 4.05)	0.49	1.15 (0.79, 1.66)	0.46
>53	1.05 (0.70, 1.58)	0.81	1.41 (0.48, 4.08)	0.53	1.07 (0.74, 1.56)	0.72
	Global test	0.66	Global test	0.56	Global test	0.42

Odds ratios (OR) and 95% confidence intervals (CI) were computed using logistic regression and adjusted for sex, age in quartiles, ever cigarette smoking, and dataset. ORs compare the odds of carrying the rs4998386-CT or TT genotypes (outcome) in coffee drinkers (exposure) to the odds of carrying the rs4998386-CT/TT genotypes in never coffee drinkers among controls (France, Denmark, Seattle-US, N = 2494; Rochester, N = 315; pooled analysis, N = 2809).

Taken altogether, these findings are not in favor of an interaction between rs4998386 and coffee drinking for the risk of PD.

## Discussion

In this large data pooling effort across multiple sites in the US and Europe, we found no evidence of an interaction between coffee intake and *GRIN2A*-rs4998386 in PD as previously reported [7], even though we included a similar number of cases and controls as the replication phase and more than twice as many participants as the discovery phase of the original study [7]. We performed extensive sensitivity analyses, in which we considered alternative definitions of coffee consumption, applied different statistical approaches, and performed stratified analyses that demonstrated the robustness of our lack of replication of the interaction between coffee intake and *GRIN2A*-rs4998386 in PD.

There are several possible explanations for our lack of replication. First, one could argue that the approach of Hamza et al. [7] is not specifically targeted at identifying gene–environment interactions: for the genome-wide discovery phase, they used the 2-df Kraft test, i.e., a test that combines marginal and interaction effects and was originally presented as a “tool for large-scale association scans where the true gene–environment interaction model is unknown” [6]. For their replication, Hamza et al. [7] specifically focused on the rs4998386-PD association among heavy coffee drinkers, which was genome-wide significant in their pooled analyses of discovery and replication data (OR = 0.51, p = 7×10^−8^); however, the test for the interaction between rs4998386 and coffee was not genome-wide significant (OR = 0.51, p = 3×10^−5^). Second, the interaction reported by Hamza et al. [7] resulted in part from a highly significant association between coffee consumption and rs4998386 among controls. Interestingly, this is the only situation where the case-only approach is less efficient than traditional case-control studies to identify gene–environment interactions [9]. The interpretation of this pattern of association in the Hamza et al. [7] study is not straightforward: while controls who carried the rs4998386-T allele were heavier coffee drinkers than noncarriers, there was a nonsignificant association between rs4998386-T and coffee in the opposite direction among PD patients, therefore suggesting that *GRIN2A* may play a role in coffee drinking behaviour with opposite effects in healthy subjects and PD cases. In contrast, we found no association between coffee drinking and rs4998386 among population controls included in the present study. This is supported by a meta-analysis of GWAS on coffee intake from eight Caucasian cohorts (n = 18,176) that found no association between the number of cups of coffee per day and *GRIN2A*-rs4998386 in healthy subjects (beta regression coefficient per one T allele = 0.0105, SE = 0.0165, p = 0.52; I^2^ = 18%, p_heterogeneity_ = 0.41; personal communication [10]). Third, PD patients included in the Hamza et al. [7] study were younger than those included in the present analysis. However, we found no evidence of interaction in analyses restricted to younger PD patients and controls. Fourth, Hamza et al. [7] used dataset-specific cutoffs to define coffee variables; this approach combines participants from separate datasets with different exposure levels in the same category and the resulting ORs do not have a simple interpretation. Our results were sensitive to the way coffee consumption data were categorized, as interaction estimates from analyses based on our main definition and those based on Hamza et al. [7] were not comparable; it is therefore possible that findings from Hamza et al. [7] may be sensitive to the way coffee data were categorized for their analyses.

According to our power calculations, our study was well powered to identify an interaction of the size estimated by Hamza et al. [7] or even weaker. The case-only approach, a method with increased statistical power to detect gene–environment interactions compared to traditional case-control analyses, relies on the assumption of gene–environment independence among controls [11]. In our study, rs4998386 was not associated with coffee consumption among controls and the case-only approach also did not identify a statistically significant gene–environment interaction; moreover, the interaction estimate was not in the same direction as reported by Hamza et al. [7]. Although the number of controls included in the present study was sufficient to detect an association between rs4998386 and coffee among controls of the size estimated based on the data from Hamza et al. [7] (Table S1), it could be argued that it was insufficient to detect a much weaker association. For this reason, we also implemented an empirical Bayes method that allows relaxing the gene–environment independence assumption while still maintaining increased efficiency compared to a traditional case-control analysis [12], and we also failed to detect an interaction using this approach.

The datasets included in the present analysis have considerable strengths. Notably, three of them were population-based with controls representative of the underlying population from which the cases arose. We included participants from various regions characterized by a wide range of coffee consumption behaviors, with a particularly high coffee consumption in the study from Denmark. These studies had a variety of designs and all failed to confirm the interaction. Most PD patients were clinically evaluated by movement disorders specialists in a standardized way in three of the studies. Finally, we used several analytic methods that all produced consistent results.

There are also limitations to this analysis. Three of the studies included prevalent PD patients; however, there was no evidence of interaction in those with shorter disease duration. Patients were not clinically assessed as part of the study in Denmark; however, PD patients were followed at neurological centers and an extensive effort was made to standardize diagnoses based on the review of the complete medical records [13]. The inverse association between coffee and PD was weaker in the Seattle-US dataset than in the European dataset, but this is unlikely to bias interaction odds ratios [14]. Coffee and PD were not inversely associated in the Rochester-US dataset, which is likely due to its use of sibling controls; this design may be less efficient to examine associations with environmental factors (because of overmatching), but it has been shown that they provide unbiased estimates of gene–environment interactions and have, in fact, increased power to detect them compared to traditional study designs [15]. Finally, we included only cases and controls of self-reported non-Hispanic Caucasian race/ethnicity and our analyses were adjusted for and stratified by the dataset, but we cannot exclude the possibility that more subtle within-study population substructure may influence our results. However, it is very unlikely that this may account for our negative findings for several reasons: (i) The frequency of rs4998386 genotypes was comparable across all studies. Hence, the minor allele frequency does not appear to vary substantially across non-Hispanic Caucasians from different countries. (ii) One of the four studies included affected PD cases and their unaffected sibs; this design is not at risk of bias due to population stratification. (iii) In this paper, our main focus is the estimate of the GRIN2A-by-coffee interaction. Previous work shows that that if there is no association between the genetic and the environmental factor within ethnic groups, the unadjusted (for population stratification) interaction estimate is unbiased [16]. As there was no association between rs4998386 and coffee in any of the datasets, it is therefore unlikely that population substructure has a major impact on our results.

In summary, our results strongly suggest that *GRIN2A*-rs4998386 does not interact with coffee for the risk of PD. Future studies of PD, coffee consumption, and genes are of continued interest to improve our understanding of whether the association between PD and coffee is truly causal, and if so, what are the underlying pathophysiological mechanisms. Such investigations may benefit substantially by considering how the interaction manifests, i.e., whether it is driven by cases or controls. The coming years will likely see an increasing number of papers on gene–environment interactions at the genome-wide scale which pose important methodological challenges. Our findings underline the need for a careful assessment of the findings of such studies.

## Methods

### Ethics statement

Written informed consent was obtained from all subjects, and the study protocol was approved by the UCLA Institutional Review Board, the Danish Data Protection Agency, the ethics committee of Copenhagen, the ethics committee of the Pitié Salpêtrière University Hospital, the Institutional Review Boards at the University of Washington and Group Health Cooperative, and the Institutional Review Board of the Mayo Clinic (Rochester, MN).

### Subjects

#### France

A population-based case-control study was performed within a health insurance system (*Mutualité Sociale Agricole*, MSA). Patients (18–80 years) from five French districts who were treated for PD were included (2006–2007) [17]. They were examined by neurologists and PD was diagnosed using standard criteria [18]. Two controls per case were randomly drawn from the electronic list of all MSA members and individually matched on age, sex, and district of residency. The reference year was the year of PD onset in cases and the same year in matched controls. The participation rate was similar in cases (82%) and controls (77%). We excluded 31 cases and 62 controls without a DNA sample or of non-European ancestry, leaving 300 cases and 598 controls for the analyses.

#### Denmark

PD patients treated at ten large neurological centers in Denmark were identified in the Danish National Hospital Register files (1996–2009), which include information since 1977 on all hospitalizations in Denmark, and matched on birth year and sex to 5–10 density sampled controls selected from the Danish Central Population Registry at time of case identification. From among 2,762 putative eligible PD cases, 179 were excluded from recruitment owing to lack of a PD diagnosis after an initial medical record review by medically trained research staff supervised by a movement disorder specialist, leaving 2,583 patients to be contacted. Of these, 497 (19%) declined participation, and the diagnosis of idiopathic PD could not be confirmed using standard diagnostic criteria for another 273 putative patients [13]. The remaining 1,813 idiopathic PD cases provided exposure data by questionnaire and interview, of whom 1,575 (87%) also provided DNA samples for genotyping. Out of 3,626 eligible controls, 1,887 completed an interview and questionnaire and 1,607 (85%) provided a DNA sample. 287 cases and 213 controls had missing information for either coffee drinking or smoking, leaving 1,288 cases and 1,394 controls for the analyses. The reference date for exposure assessment was the occurrence of the first cardinal (motor) symptom or the first date of PD diagnosis from the medical records, and controls were assigned the date of their respective matched cases.

#### Seattle-US

Newly diagnosed PD cases were identified in a population-based case-control study conducted in western Washington State at Group Health Cooperative (GHC), a health maintenance organization, and the University of Washington Neurology Clinic in Seattle (1992–2008) [19], [20]. All diagnoses were confirmed by a neurologist or verified by a team of neurologists by consensus chart review [18]. Cases were enrolled within four years of diagnosis (most within two years), and all had a MMSE score ≥24. Controls were neurologically normal (no history of multiple sclerosis, Alzheimer's disease, or other neurodegenerative disorder, MMSE score ≥24), enrolled in GHC, and frequency-matched to cases by sex, age, race and ethnicity, clinic, and year of GHC enrollment. 386 cases and 502 controls who were non-Hispanic Caucasians with genotyping, coffee, and smoking data were included in the analyses.

#### Rochester-US

This family-based dataset consists of 443 discordant sibling pairs, such that each sibling pair has one member affected with PD and one unaffected. Cases residing in Minnesota or one of the surrounding states (Wisconsin, Iowa, South Dakota, and North Dakota) were enrolled (1996–2004) at the Department of Neurology of the Mayo Clinic (Rochester, MN). All cases underwent a standardized clinical assessment performed by a neurologist; PD was diagnosed using standard criteria [18]. Cases provided a genealogical history, and, when permitted, available siblings were contacted for a telephone interview to exclude parkinsonism via a validated screening instrument. Cases were matched to a single participating sibling without parkinsonism, first by sex (when possible) and then by closest age [21]. Exposure data were obtained by direct (or proxy for incapacitated subjects) interview using a structured questionnaire administered via telephone by trained research assistants blinded to case-control status [22]. Both genotype and relevant exposures were available for 315 pairs.

### Genotyping

The source of DNA was saliva (Oragene) for France and Denmark, blood (86%) and buccal specimens (14%) for Seattle-US, and blood for Rochester-US.

SNP rs4998386 was genotyped in the French (call rate, 97%), Seattle-US (call rate, 97%) and Danish (call rate, 96%) datasets using allelic discrimination assays based on TaqMan chemistry according to the manufacturer's protocol (Life Technologies, Inc). For the genotyping of the French samples, six DNA samples from the PEG dataset included in the study of Hamza et al. [7] were included on each plate (concordance rate, 100%). For the Danish samples, each plate included 5% HapMap CEU (Northern/Western Europe ancestry) control samples (concordance rate, 100%).

The Rochester-US dataset contained individual-level genotype data from the “Mayo-Perlegen Linked Efforts to Accelerate Parkinson's Solutions (LEAPS) Collaboration” [21]. Genotyping was performed using a Perlegen platform (198,345 SNPs). Data cleaning was performed with the PLINK toolset v1.07 (http://pngu.mgh.harvard.edu/~purcell/plink/) as previously described [2]. Briefly, samples were cleaned on the basis of genotyping efficiency and quality by excluding all SNPs with a minor allele frequency (MAF)<0.01, missing rates >2%, or HWE violations (P<1×10^−6^); samples with genotyping efficiency <95% were excluded. This resulted in 149,817 analyzable SNPs (433 PD cases, 428 controls). rs4998386 was not genotyped and was imputed as follows: uncovered autosomal SNPs were imputed on a genome-wide scale from the cleaned dataset using the IMPUTE program v2.0 (http://mathgen.stats.ox.ac.uk/impute/impute_v2.html) and the precompiled HapMap 3 (release #2) and 1000 Genomes CEU+TSI (Pilot 1) panels (obtained on June 2nd, 2010). Individual-level genotypes were assigned according to the genotype called with 0.9 or greater posterior call probability, or coded as missing if the posterior call probability fell below 0.9. Imputed datasets were cleaned following the same thresholds as outlined above. The final dataset consisted of 735,746 SNPs (429 PD cases, 427 controls), including rs4998386.

### Coffee intake

Analyses were based on caffeinated coffee consumption, as for three out of the four datasets included in Hamza et al. [7]. All studies assessed whether participants had ever been coffee drinkers. The French, Danish, and Rochester-US studies collected information on the usual number of cups of coffee per day that participants drank during several periods of life, thus allowing us to compute an average number of coffee cups per day and a cumulative number of coffee cupyears (number of cups per day multiplied by the number of years); only exposures occurring prior to PD onset in cases or the reference date in controls were considered. The Seattle-US study collected information on the typical lifetime number of coffee cups per day, but not on duration of coffee drinking; we used data from a PD case-control study conducted in California (PEG) and included in Hamza et al. [7] to impute average duration of coffee drinking. Among several covariates (PD disease status, smoking, age, sex, number of coffee cups per day), the main determinants of duration of coffee drinking were age and number of coffee cups per day; we used these covariates to impute duration of coffee drinking using linear regression for this study.

For our main analyses, we used four different definitions of coffee intake: (i) never versus ever coffee drinking; (ii) number of cups per day in four classes (never, 1, 2, ≥3); (iii) cupyears (never, [0–65], [65–130], [130–200], >200); (iv) years of coffee drinking (never, [0–37], [37–45], [45– 53], >53); cutoffs were chosen a priori, based on the inspection of the distributions of the variables in each dataset, so that there was a sufficient number of exposed subjects in each category in all studies.

In sensitivity analyses, we used the same approach as Hamza et al. [7]: median cupyears was determined among controls (excluding those with zero intake) in each dataset separately and used to distinguish light (never, ≤median) from heavy (>median) drinkers; in addition, quartiles of cupyears were defined among controls from each dataset using the full range (from zero to maximum intake). We did not use this approach as our primary method, because it combines in the same category participants from different datasets with different exposure levels and creates difficulties for the interpretation of results. In addition, never coffee drinkers may have different characteristics compared to coffee drinkers, but are not considered as a distinct category. Additionally, we performed sensitivity analyses for the Seattle-US dataset using caffeine intake from seven food and beverage sources as the exposure variable [23].

### Power calculations

Power calculations were performed using the Quanto Software [24].

#### Case-control analysis

Based on the following parameters (minor allele frequency, MAF: 10%; ever coffee drinking: 80%; marginal genetic OR: 1.0; marginal coffee OR: 0.75; n_cases_ = 2289, n_controls_ = 2809), our study had a power of 98.8% to detect an interaction OR of 0.5 (as estimated by the original study [7]) at the 0.05 two-sided level; power was still adequate (86.7%) to detect a weaker interaction OR of 0.6. Assuming a 40% exposure frequency for heavy versus light coffee drinking, the power to detect an interaction OR of 0.5 was 99.3% and it was 91.0% to detect an interaction OR of 0.6.

#### Case-only analysis

Our study had a power of 99.9% to detect an interaction OR of 0.5; power was still adequate (85.0%) to detect a weaker interaction OR of 0.7. If we assumed a 40% exposure frequency (heavy versus light coffee drinking), the power to detect an interaction OR of 0.5 was 99.9% and it was 86.6% to detect an interaction OR of 0.7.

### Statistical analysis

Only non-Hispanic Caucasian subjects were included in the analysis as there were very few subjects from other racial and ethnic groups in all studies. We checked that rs4998386 genotypes were in HWE among controls from each dataset using an exact test (p≥0.05). All analyses were first performed independently for each dataset. For the French, Danish, and Seattle-US studies, we computed ORs and 95% confidence intervals (CI) using unconditional logistic regression adjusted for age (in quartiles) and sex; we broke the matching for the French and Danish studies as some participants did not provide DNA. For the Rochester-US dataset, we used conditional logistic regression to take into account the fact that cases and controls were related. All analyses were also adjusted for ever cigarette smoking. Second, we obtained pooled OR estimates for the French, Danish, and Seattle-US studies by using unconditional logistic regression adjusted for age (in quartiles), sex, ever cigarette smoking, and dataset; we did not include the Rochester-US dataset at this stage due to the difference in study design. Finally, we combined the four studies in a single analysis by using an approach that allows pooling of individual data from matched and unmatched case-control studies [25], [26].

We examined the marginal association of rs4998386 and coffee with PD. Since TT homozygotes were very rare (<1% of controls in all studies), we used a dominant model of inheritance (at least one T-allele versus none); in sensitivity analyses, we excluded TT-homozygotes to check for the robustness of our results [7].

To investigate the interaction between rs4998386 and coffee, we used a variety of approaches. We estimated the individual and joint effects of rs4998386 (dominant coding) and coffee and performed a statistical test of interaction by including multiplicative terms between rs4998386 and each category of coffee drinking in the models while retaining all respective main effects [27]. A global test of interaction was performed by comparing the log-likelihood of this model with that of a model without interaction terms; this approach is preferable compared to including interactions with linear continuous variables that may lead to biased interaction estimates [28]. We tested for interactions on a multiplicative scale as in the original paper [7]; when at least one exposure is “preventive” (e.g., coffee), multiplicative statistical models are appropriate according to several causal models [29]. Interactions were also estimated through an empirical Bayes method that allows relaxing the gene–environment independence hypothesis among controls and is usually more powerful than the standard analysis [12]; this approach is only available for unmatched case-control data and was not implemented for the Rochester-US dataset.

Finally, we studied the association between rs4998386 and coffee separately among cases and controls using unconditional logistic regression adjusted for age (in quartiles), sex, ever cigarette smoking, and dataset. Under the hypothesis of gene-environment independence among controls (i.e., rs4998386 is not associated with coffee drinking behavior), a significant association between rs4998386 and coffee among cases indicates an interaction. This approach is usually more powerful than a traditional case-control analysis; however, if the hypothesis of gene–environment independence among controls does not hold, interaction ORs are biased and type 1 error is inflated [11].

In sensitivity analyses, we performed analyses stratified by sex. Since participants included in the present study were on average older than those included in the original paper [7], we also performed analyses stratified by median age. We assessed whether disease duration had an influence on our findings by performing analyses by disease duration (<5 years, ≥5 years). We also checked whether adjusting for MMSE (available for the French and Seattle-US datasets) or packyears of smoking had an impact on our findings.

Conditional and unconditional logistic regression and empirical Bayes analyses were performed with R, v3.01 (R-Foundation for Statistical Computing, Vienna, Austria).

## Supporting Information

Table S1 Re-analysis of the relation between and coffee in cases-only and controls-only based on data from Hamza et al.
(DOCX)

Table S2 Participants' characteristics by dataset.
(DOCX)

Table S3 Marginal association of coffee drinking and *GRIN2A*-rs4998386 with Parkinson disease.
(DOCX)

Table S4 Joint distribution of *GRIN2A*-rs4998386 and variables related to coffee drinking in cases and controls by dataset.
(DOCX)

Table S5 Independent and joint effects of coffee drinking and *GRIN2A*-rs4998386 for Parkinson disease by dataset.
(DOCX)

Table S6 Pooled analysis of independent and joint effects of coffee drinking and *GRIN2A*-rs4998386 for Parkinson disease: alternative definitions of coffee drinking.
(DOCX)

Table S7 Pooled analysis of independent and joint effects of coffee drinking and *GRIN2A*-rs4998386 for Parkinson disease: empirical Bayes approach for estimating interactions.
(DOCX)
