# Supplementary material for: Lack of Replication of the GRIN2A-by-Coffee Interaction in Parkinson Disease
Source: PLoS Genet. 2014 Nov 20;10(11):e1004788. doi: 10.1371/journal.pgen.1004788 (PMC4238979; doi:10.1371/journal.pgen.1004788)
Supplement: Table S1 — Re-analysis of the relation between and coffee in cases-only and controls-only based on data from Hamza et al. (DOCX) [file pgen.1004788.s001.docx]

Table S1. Re-analysis of the relation between and coffee in cases-only and controls-only based on data from Hamza *et al*.^a^

| Study | rs4998386 | Coffee |  |  | Cases (N) | Controls (N) |
| --- | --- | --- | --- | --- | --- | --- |
| NGRC | CC | Light |  |  | 786 | 433 |
|  | CC | Heavy |  |  | 441 | 283 |
|  | CT-TT | Light |  |  | 160 | 111 |
|  | CT-TT | Heavy |  |  | 71 | 104 |
|  |  |  |  |  | OR^b^=0.79 (0.58, 1.07) | OR^b^=1.43 (1.06, 1.95) |
|  |  |  |  |  | p=0.13 | p=0.021 |
|  |  |  |  |  |  |  |
| PEG | CC | Light |  |  | 154 | 155 |
|  | CC | Heavy |  |  | 80 | 94 |
|  | CT-TT | Light |  |  | 30 | 33 |
|  | CT-TT | Heavy |  |  | 16 | 28 |
|  |  |  |  |  | OR^b^=1.03 (0.53, 1.99) | OR^b^=1.40 (0.80, 2.46) |
|  |  |  |  |  | p=0.94 | p=0.24 |
|  |  |  |  |  |  |  |
| PAGE | CC | Light |  |  | 235 | 623 |
|  | CC | Heavy |  |  | 199 | 576 |
|  | CT-TT | Light |  |  | 55 | 115 |
|  | CT-TT | Heavy |  |  | 36 | 160 |
|  |  |  |  |  | OR^b^=0.77 (0.49, 122) | OR^b^=1.50 (1.15, 1.96) |
|  |  |  |  |  | p=0.27 | p=0.0025 |
|  |  |  |  |  |  |  |
| HIHG | CC | Light |  |  | 118 | 74 |
|  | CC | Heavy |  |  | 51 | 36 |
|  | CT-TT | Light |  |  | 29 | 12 |
|  | CT-TT | Heavy |  |  | 11 | 11 |
|  |  |  |  |  | OR^b^=0.88 (0.41, 1.89) | OR^b^=1.88 (0.76, 4.68) |
|  |  |  |  |  | p=0.74 | p=0.17 |
|  |  |  |  |  |  |  |
| Pooled analysis | |  |  |  | OR^c^=0.82 (0.65, 1.03) | OR^c^=1.48 (1.23, 1.78) |
|  |  |  |  |  | p=0.080 | P<0.0001 |

Odds ratios (OR) compare the odds of carrying the rs4998386-CT or TT genotypes (outcome) in heavy coffee drinkers (exposure) to the odds of carrying the rs4998386-CT/TT genotypes in light coffee drinkers, separately in cases and controls

^a^ Hamza TH, Chen H, Hill-Burns EM, Rhodes SL, Montimurro J, et al. (2011) Genome-wide gene-environment study identifies glutamate receptor gene GRIN2A as a Parkinson’s disease modifier gene via interaction with coffee. PLoS genetics 7: e1002237. doi:10.1371/journal.pgen.1002237.

^b^ ORs and 95% confidence intervals computed using unconditional logistic regression.

^c^ ORs and 95% confidence intervals computed using unconditional logistic regression and adjusted for dataset.
