# Supplementary material for: Lack of Replication of the GRIN2A-by-Coffee Interaction in Parkinson Disease
Source: PLoS Genet. 2014 Nov 20;10(11):e1004788. doi: 10.1371/journal.pgen.1004788 (PMC4238979; doi:10.1371/journal.pgen.1004788)
Supplement: Table S2 — Participants' characteristics by dataset. (DOCX) [file pgen.1004788.s002.docx]

Table S2. Participants’ characteristics by dataset.

|  | France | |  | Denmark | |  | Seattle-US | |  | Rochester-US | |
| --- | --- | --- | --- | --- | --- | --- | --- | --- | --- | --- | --- |
| Characteristics | Controls | Cases |  | Controls | Cases |  | Controls | Cases |  | Controls | Cases |
| N | 598 | 300 |  | 1394 | 1288 |  | 502 | 386 |  | 315 | 315 |
| Mean age at study (SD) | 72.7 (6.6) | 72.7 (6.6) |  | 68.3 (8.6) | 67.7 (8.3) |  | 68.7 (8.6) | 66.3 (10.2)*** |  | 64.3 (11.0) | 64.8 (10.4) |
| Women | 245 (41.0) | 126 (42.0) |  | 535 (38.4) | 502 (39.0) |  | 182 (36.3) | 137 (35.5) |  | 152 (48.3) | 190 (60.3)*** |
| Ever smoker | 194 (32.4) | 72 (24.0)** |  | 901 (64.6) | 657 (51.0)*** |  | 279 (55.6) | 167 (43.3)*** |  | 172 (54.6) | 155 (49.2)** |
| *GRIN2A*- rs4998386^a^ |  |  |  |  |  |  |  |  |  |  |  |
| CC | 492 (82.3) | 242 (80.7) |  | 1094 (78.5) | 1042 (80.9) |  | 418 (83.3) | 302 (78.2) |  | 244 (77.5) | 247 (78.4) |
| CT | 101 (16.9) | 56 (18.7) |  | 286 (20.5) | 232 (18) |  | 81 (16.1) | 77 (19.9) |  | 68 (21.6) | 68 (21.6) |
| TT | 5 (0.8) | 2 (0.7) |  | 14 (1) | 14 (1.1) |  | 3 (0.6) | 7 (1.8) |  | 3 (0.9) | 0 (0.0) |
| Ever coffee drinking | 490 (81.9) | 227 (75.7)* |  | 1356 (97.3) | 1206 (93.6)*** |  | 374 (74.5) | 282 (73.1) |  | 263 (83.5) | 250 (79.4) |
| Cupyears |  |  |  |  |  |  |  |  |  |  |  |
| Never | 108 (18.1) | 73 (24.3) |  | 38 (2.7) | 82 (6.4) |  | 128 (25.5) | 104 (26.9) |  | 52 (16.5) | 65 (20.6) |
| ]0, 65] | 177 (29.6) | 72 (24.0)* |  | 162 (11.6) | 199 (15.5)*** |  | 119 (23.7) | 121 (31.3) |  | 83 (26.3) | 68 (21.6) |
| ]65, 130] | 150 (25.1) | 88 (29.3) |  | 332 (23.8) | 331 (25.7)*** |  | 64 (12.7) | 55 (14.2) |  | 74 (23.5) | 75 (23.8) |
| ]130, 200] | 123 (20.6) | 47 (15.7)** |  | 329 (23.6) | 285 (22.1)*** |  | 112 (22.3) | 63 (16.3) |  | 50 (15.9) | 46 (14.6) |
| >200 | 40 (6.7) | 20 (6.7) |  | 533 (38.2) | 391 (30.4)*** |  | 79 (15.7) | 43 (11.1) |  | 56 (17.8) | 61 (19.4) |
| Cups per day |  |  |  |  |  |  |  |  |  |  |  |
| Never | 108 (18.1) | 73 (24.3) |  | 38 (2.7) | 82 (6.4) |  | 128 (25.5) | 104 (26.9) |  | 52 (16.5) | 65 (20.6) |
| 1 | 168 (28.1) | 77 (25.7) |  | 89 (6.4) | 134 (10.4) |  | 115 (22.9) | 113 (29.3) |  | 113 (35.9) | 96 (30.5) |
| 2 | 146 (24.4) | 75 (25.0) |  | 263 (18.9) | 260 (20.2)*** |  | 171 (34.1) | 116 (30.1) |  | 49 (15.6) | 55 (17.5) |
| ≥3 | 176 (29.4) | 75 (25.0)* |  | 1004 (72.0) | 812 (63.0)*** |  | 88 (17.5) | 53 (13.7) |  | 101 (32.1) | 99 (31.4) |
| Years of coffee drinking |  |  |  |  |  |  |  |  |  |  |  |
| Never | 108 (18.1) | 73 (24.3) |  | 38 (2.7) | 82 (6.4) |  | 128 (25.5) | 104 (26.9) |  | 52 (16.5) | 65 (20.6) |
| ]0, 37] | 92 (15.4) | 38 (12.7)* |  | 410 (29.4) | 415 (32.2)*** |  | 70 (13.9) | 78 (20.2) |  | 54 (17.1) | 51 (16.2) |
| ]37, 45] | 124 (20.7) | 52 (17.3)* |  | 345 (24.7) | 307 (23.8)*** |  | 97 (19.3) | 73 (18.9) |  | 82 (26) | 80 (25.4) |
| ]45, 53] | 141 (23.6) | 72 (24.0) |  | 341 (24.5) | 292 (22.7)*** |  | 56 (11.2) | 42 (10.9) |  | 55 (17.5) | 40 (12.7) |
| >53 | 133 (22.2) | 65 (21.7) |  | 260 (18.7) | 192 (14.9)*** |  | 151 (30.1) | 89 (23.1) |  | 72 (22.9) | 79 (25.1) |
| Cupyears^b^ |  |  |  |  |  |  |  |  |  |  |  |
| Light | 354 (59.2) | 186 (62.0) |  | 725 (52) | 785 (60.9) |  | 311 (62.0) | 279 (72.3) |  | 186 (59.0) | 185 (58.7) |
| Heavy | 244 (40.8) | 114 (38.0) |  | 669 (48) | 503 (39.1)*** |  | 191 (38.0) | 107 (27.7)* |  | 129 (41.0) | 130 (41.3) |
| Quartiles of cupyears^b^ |  |  |  |  |  |  |  |  |  |  |  |
| [0%,25%] | 150 (25.1) | 90 (30.0) |  | 355 (25.5) | 443 (34.4) |  | 128 (25.5) | 104 (26.9) |  | 75 (23.8) | 91 (28.9) |
| ]25%, 50%] | 150 (25.1) | 65 (21.7) |  | 346 (24.8) | 325 (25.2)* |  | 139 (27.7) | 137 (35.5) |  | 83 (26.3) | 56 (17.8) |
| ]50%,75%] | 148 (24.7) | 86 (28.7) |  | 346 (24.8) | 282 (21.9)*** |  | 140 (27.9) | 87 (22.5) |  | 76 (24.1) | 86 (27.3) |
| ]75%,100%] | 150 (25.1) | 59 (19.7)* |  | 347 (24.9) | 238 (18.5)*** |  | 95 (18.9) | 58 (15.0) |  | 81 (25.7) | 82 (26.0) |
| Mean age at diagnosis (SD) | - | 66.9 (7.5) |  | - | 62.3 (9.1) |  | - | 65.5 (10.2) |  | -- | 61.2 (11.1) |
| Mean age at onset (SD) | - | 66.3 (7.6) |  | - | 60.8 (9.3) |  | - | NA |  | -- | 59.7 (11.1) |
| Family history of PD | 22 (3.7) | 35 (11.7)*** |  | 78 (5.6) | 177 (13.7)*** |  | 21 (5.3) | 34 (11.0)* |  | -- | 62 (19.8) |
| Mean MMSE (SD) | 26.0 (3.3) | 24.9 (3.8)*** |  | NA | NA |  | 28.6 (1.3) | 28.4 (1.5)** |  | NA | 28.7 (1.8) |

Numbers are n's and percentages unless otherwise stated. NA, not available. PD, Parkinson's disease. SD, standard deviation. Age- and sex-adjusted P-values were computed using unconditional logistic regression, except for the Rochester-US dataset for which we used conditional logistic regression. * p<0.05, ** p<0.01, *** p<0.001.

^a^ The frequency of the T-allele among controls was: France, 9.3%; Denmark, 11.3%; Seattle-US, 8.7%; Rochester-US, 11.8%. rs4998386 was in HWE among controls: France, p=0.99; Denmark, p=0.42; Seattle-US, p=0.99; Rochester-US, p=0.45.^b^ According to Hamza et al. (2011) Genome-wide gene-environment study identifies glutamate receptor gene GRIN2A as a Parkinson’s disease modifier gene via interaction with coffee. PLoS genetics 7: e1002237. doi:10.1371/journal.pgen.1002237.
