# Supplementary material for: Lack of Replication of the GRIN2A-by-Coffee Interaction in Parkinson Disease
Source: PLoS Genet. 2014 Nov 20;10(11):e1004788. doi: 10.1371/journal.pgen.1004788 (PMC4238979; doi:10.1371/journal.pgen.1004788)
Supplement: Table S3 — Marginal association of coffee drinking and GRIN2A-rs4998386 with Parkinson disease. (DOCX) [file pgen.1004788.s003.docx]

Table S3. Marginal association of coffee drinking and *GRIN2A*-rs4998386 with Parkinson’s disease.

|  |  |  | France, Denmark,  Seattle-US | |  | Rochester-US | |
| --- | --- | --- | --- | --- | --- | --- | --- |
|  |  |  | OR (95% CI)^a^ | p |  | OR (95% CI)^b^ | p |
| **Coffee** | | | | | | | |
| Ever *vs* never | |  |  |  |  |  |  |
|  | Never |  | 1.00 (Ref.) | - |  | 1.00 (Ref.) | - |
|  | Ever |  | 0.73 (0.60, 0.88) | 0.001 |  | 1.04 (0.64, 1.67) | 0.89 |
|  |  |  |  |  |  |  |  |
| Cups per day | |  |  |  |  |  |  |
|  | Never |  | 1.00 (Ref.) |  |  | 1.00 (Ref.) | - |
|  | 1 cup |  | 0.93 (0.74, 1.17) | 0.55 |  | 0.94 (0.57, 1.54) | 0.79 |
|  | 2 cups |  | 0.73 (0.59, 0.91) | 0.005 |  | 1.27 (0.67, 2.40) | 0.46 |
|  | ≥ 3 cups |  | 0.60 (0.48, 0.74) | <0.001 |  | 1.20 (0.69, 2.08) | 0.53 |
|  |  |  | Trend | <0.001^c^ |  | Trend | 0.34^c^ |
|  | |  |  |  |  |  |  |
| Cupyears | |  |  |  |  |  |  |
|  | Never |  | 1.00 (Ref.) |  |  | 1.00 (Ref.) |  |
|  | ]0, 65] |  | 0.85 (0.68, 1.07) | 0.17 |  | 0.91 (0.54, 1.52) | 0.71 |
|  | ]65, 130] |  | 0.79 (0.63, 0.98) | 0.035 |  | 1.14 (0.64, 2.02) | 0.67 |
|  | ]130, 200] |  | 0.63 (0.50, 0.79) | <0.001 |  | 1.07 (0.55, 2.07) | 0.84 |
|  | >200 |  | 0.60 (0.48, 0.76) | <0.001 |  | 1.38 (0.74, 2.57) | 0.31 |
|  |  |  | Trend | <0.001^c^ |  | Trend | 0.17^c^ |
|  | | | |  |  |  |  |
| Years of coffee drinking | | | |  |  |  |  |
|  | Never |  | 1.00 (Ref.) |  |  | 1.00 (Ref.) |  |
|  | ]0, 37] |  | 0.79 (0.62, 0.99) | 0.041 |  | 1.08 (0.61, 1.93) | 0.79 |
|  | ]37, 45] |  | 0.71 (0.56, 0.89) | 0.003 |  | 1.11 (0.63, 1.95) | 0.72 |
|  | ]45, 53] |  | 0.74 (0.59, 0.93) | 0.009 |  | 0.80 (0.42, 1.53) | 0.50 |
|  | >53 |  | 0.70 (0.55, 0.88) | 0.002 |  | 1.19 (0.58, 2.42) | 0.63 |
|  |  |  | Trend | <0.001^c^ |  | Trend | 0.84^c^ |
|  | | | | | | | |
| ***GRIN2A*-rs4998386** | | | | | | | |
|  | CC |  | 1.00 (Ref.) |  |  | 1.00 (Ref.) |  |
|  | CT, TT |  | 0.98 (0.84, 1.14) | 0.82 |  | 0.87 (0.50, 1.51) | 0.62 |

^a^ Odds ratios (OR) and 95% confidence intervals (CI) computed using unconditional logistic regression and adjusted for sex, age in quartiles, ever cigarette smoking, and dataset (1974 cases, 2494 controls).

^b^ Odds ratios (OR) and 95% confidence intervals (CI) computed using conditional logistic regression and adjusted for sex, age in quartiles, and ever cigarette smoking (315 cases, 315 controls).

^c^ Trend test: we defined ordinal variables by assigning its median value to each category.
