# Supplementary material for: Lack of Replication of the GRIN2A-by-Coffee Interaction in Parkinson Disease
Source: PLoS Genet. 2014 Nov 20;10(11):e1004788. doi: 10.1371/journal.pgen.1004788 (PMC4238979; doi:10.1371/journal.pgen.1004788)
Supplement: Table S4 — Joint distribution of GRIN2A-rs4998386 and variables related to coffee drinking in cases and controls by dataset. (DOCX) [file pgen.1004788.s004.docx]

Table S4. Joint distribution of *GRIN2A*- rs4998386 and variables related to coffee drinking in cases and controls by dataset.

|  |  | France | |  | Denmark | |  | Seattle-US | |  | Rochester-US | |
| --- | --- | --- | --- | --- | --- | --- | --- | --- | --- | --- | --- | --- |
| *GRIN2A*- |  | Controls | Cases |  | Controls | Cases |  | Controls | Cases |  | Controls | Cases |
| rs4998386 | Coffee | N (%) | N (%) |  | N (%) | N (%) |  | N (%) | N (%) |  | N (%) | N (%) |
| CC | Never | 86 (14.4) | 55 (18.3) |  | 36 (2.6) | 68 (5.3) |  | 107 (21.3) | 85 (22.0) |  | 42 (13.3) | 54 (17.1) |
| CC | Ever | 406 (67.9) | 187 (62.3) |  | 1058 (75.9) | 974 (75.6) |  | 311 (62.0) | 217 (56.2) |  | 202 (64.1) | 193 (61.3) |
| CT+TT | Never | 22 (3.7) | 18 (6.0) |  | 2 (0.1) | 14 (1.1) |  | 21 (4.2) | 19 (4.9) |  | 10 (3.2) | 11 (3.5) |
| CT+TT | Ever | 84 (14.1) | 40 (13.3) |  | 298 (21.4) | 232 (18.0) |  | 63 (12.6) | 65 (16.8) |  | 61 (19.4) | 57 (18.1) |
|  |  |  |  |  |  |  |  |  |  |  |  |  |
|  | *Cupyears* |  |  |  |  |  |  |  |  |  |  |  |
| CC | Never | 86 (14.4) | 55 (18.3) |  | 36 (2.6) | 68 (5.3) |  | 107 (21.3) | 85 (22.0) |  | 42 (13.3) | 54 (17.1) |
| CC | ]0, 65] | 147 (24.6) | 61 (20.3) |  | 126 (9.0) | 160 (12.4) |  | 101 (20.1) | 91 (23.6) |  | 64 (20.3) | 54 (17.1) |
| CC | ]65, 130] | 125 (20.9) | 74 (24.7) |  | 261 (18.7) | 276 (21.4) |  | 52 (10.4) | 39 (10.1) |  | 56 (17.8) | 61 (19.4) |
| CC | ]130, 200] | 99 (16.6) | 39 (13.0) |  | 244 (17.5) | 231 (17.9) |  | 93 (18.5) | 53 (13.7) |  | 36 (11.4) | 32 (10.2) |
| CC | >200 | 35 (5.9) | 13 (4.3) |  | 427 (30.6) | 307 (23.8) |  | 65 (13.0) | 34 (8.8) |  | 46 (14.6) | 46 (14.6) |
| CT+TT | Never | 22 (3.7) | 18 (6.0) |  | 2 (0.1) | 14 (1.1) |  | 21 (4.2) | 19 (4.9) |  | 10 (3.2) | 11 (3.5) |
| CT+TT | ]0, 65] | 30 (5.0) | 11 (3.7) |  | 36 (2.6) | 39 (3.0) |  | 18 (3.6) | 30 (7.8) |  | 19 (6.0) | 14 (4.4) |
| CT+TT | ]65, 130] | 25 (4.2) | 14 (4.7) |  | 71 (5.1) | 55 (4.3) |  | 12 (2.4) | 16 (4.2) |  | 18 (5.7) | 14 (4.4) |
| CT+TT | ]130, 200] | 24 (4.0) | 8 (2.7) |  | 85 (6.1) | 54 (4.2) |  | 19 (3.8) | 10 (2.6) |  | 14 (4.4) | 14 (4.4) |
| CT+TT | >200 | 5 (0.8) | 7 (2.3) |  | 106 (7.6) | 84 (6.5) |  | 14 (2.8) | 9 (2.3) |  | 10 (3.2) | 15 (4.8) |
|  |  |  |  |  |  |  |  |  |  |  |  |  |
| CC | Light | 293 (49.0) | 151 (50.3) |  | 563 (40.4) | 647 (50.2) |  | 260 (51.8) | 214 (55.4) |  | 142 (45.1) | 150 (47.6) |
| CC | Heavy | 199 (33.3) | 91 (30.3) |  | 531 (38.1) | 395 (30.7) |  | 158 (31.5) | 88 (22.8) |  | 102 (32.4) | 97 (30.8) |
| CT+TT | Light | 61 (10.2) | 35 (11.7) |  | 162 (11.6) | 138 (10.7) |  | 51 (10.2) | 65 (16.8) |  | 44 (14.0) | 35 (11.1) |
| CT+TT | Heavy | 45 (7.5) | 23 (7.7) |  | 138 (9.9) | 108 (8.4) |  | 33 (6.6) | 19 (4.9) |  | 27 (8.6) | 33 (10.5) |
|  |  |  |  |  |  |  |  |  |  |  |  |  |
| CC | [0%,25%] | 117 (19.6) | 70 (23.3) |  | 285 (20.4) | 359 (27.9) |  | 107 (21.3) | 85 (22.0) |  | 61 (19.4) | 70 (22.2) |
| CC | ]25%, 50%] | 126 (21.1) | 55 (18.3) |  | 256 (18.4) | 273 (21.2) |  | 118 (23.5) | 103 (26.7) |  | 60 (19.1) | 49 (15.6) |
| CC | ]50%,75%] | 125 (20.9) | 73 (24.3) |  | 267 (19.2) | 218 (16.9) |  | 114 (22.7) | 67 (17.4) |  | 60 (19.1) | 69 (21.9) |
| CC | ]75%,100%] | 124 (20.7) | 44 (14.7) |  | 286 (20.5) | 192 (14.9) |  | 79 (15.7) | 47 (12.2) |  | 63 (20.0) | 59 (18.7) |
| CT+TT | [0%,25%] | 33 (5.5) | 20 (6.7) |  | 70 (5.0) | 84 (6.5) |  | 21 (4.2) | 19 (4.9) |  | 14 (4.4) | 21 (6.7) |
| CT+TT | ]25%, 50%] | 24 (4.0) | 10 (3.3) |  | 90 (6.5) | 52 (4.0) |  | 21 (4.2) | 34 (8.8) |  | 23 (7.3) | 7 (2.2) |
| CT+TT | ]50%,75%] | 23 (3.9) | 13 (4.3) |  | 79 (5.7) | 64 (5.0) |  | 26 (5.2) | 20 (5.2) |  | 16 (5.1) | 17 (5.4) |
| CT+TT | ]75%,100%] | 26 (4.4) | 15 (5.0) |  | 61 (4.4) | 46 (3.6) |  | 16 (3.2) | 11 (2.9) |  | 18 (5.7) | 23 (7.3) |
|  |  |  |  |  |  |  |  |  |  |  |  |  |

Table S4 (follows).

|  |  | France | |  | Denmark | |  | Seattle-US | |  | Rochester-US | |
| --- | --- | --- | --- | --- | --- | --- | --- | --- | --- | --- | --- | --- |
| *GRIN2A*- |  | Controls | Cases |  | Controls | Cases |  | Controls | Cases |  | Controls | Cases |
| rs4998386 | Coffee | N (%) | N (%) |  | N (%) | N (%) |  | N (%) | N (%) |  | N (%) | N (%) |
|  | *Cups per day* |  |  |  |  |  |  |  |  |  |  |  |
| CC | Never | 86 (14.4) | 55 (18.3) |  | 36 (2.6) | 68 (5.3) |  | 107 (21.3) | 85 (22.0) |  | 42 (13.3) | 54 (17.1) |
| CC | 1 | 136 (22.7) | 65 (21.7) |  | 66 (4.7) | 110 (8.5) |  | 98 (19.5) | 84 (21.8) |  | 85 (27.0) | 78 (24.8) |
| CC | 2 | 124 (20.7) | 65 (21.7) |  | 208 (14.9) | 211 (16.4) |  | 140 (27.9) | 90 (23.3) |  | 37 (11.8) | 38 (12.1) |
| CC | ≥3 | 146 (24.4) | 57 (19.0) |  | 784 (56.2) | 653 (50.7) |  | 73 (14.5) | 43 (11.1) |  | 80 (25.4) | 77 (24.4) |
| CT+TT | Never | 22 (3.7) | 18 (6.0) |  | 2 (0.1) | 14 (1.1) |  | 21 (4.2) | 19 (4.9) |  | 10 (3.2) | 11 (3.5) |
| CT+TT | 1 | 32 (5.4) | 12 (4.0) |  | 23 (1.7) | 24 (1.9) |  | 17 (3.4) | 29 (7.5) |  | 28 (8.9) | 18 (5.7) |
| CT+TT | 2 | 22 (3.7) | 10 (3.3) |  | 55 (4.0) | 49 (3.8) |  | 31 (6.2) | 26 (6.7) |  | 12 (3.8) | 17 (5.4) |
| CT+TT | ≥3 | 30 (5.0) | 18 (6.0) |  | 220 (15.8) | 159 (12.3) |  | 15 (3.0) | 10 (2.6) |  | 21 (6.7) | 22 (7.0) |
|  |  |  |  |  |  |  |  |  |  |  |  |  |
|  | *Number of years of coffee drinking* | | |  |  |  |  |  |  |  |  |  |
| CC | Never | 86 (14.4) | 55 (18.3) |  | 36 (2.6) | 68 (5.3) |  | 107 (21.31) | 85 (22.0) |  | 42 (13.3) | 54 (17.1) |
| CC | ]0, 37] | 78 (13.0) | 34 (11.3) |  | 321 (23.0) | 342 (26.6) |  | 62 (12.4) | 59 (15.3) |  | 44 (14.0) | 38 (12.1) |
| CC | ]37, 45] | 100 (16.7) | 37 (12.3) |  | 263 (18.9) | 243 (18.9) |  | 79 (15.7) | 54 (14.0) |  | 59 (18.7) | 65 (20.6) |
| CC | ]45, 53] | 121 (20.2) | 63 (21.0) |  | 260 (18.7) | 239 (18.6) |  | 48 (9.6) | 32 (8.3) |  | 43 (13.7) | 33 (10.5) |
| CC | >53 | 107 (17.9) | 53 (17.7) |  | 214 (15.4) | 150 (11.7) |  | 122 (24.3) | 72 (18.7) |  | 56 (17.8) | 57 (18.1) |
| CT+TT | Never | 22 (3.7) | 18 (6.0) |  | 2 (0.1) | 14 (1.1) |  | 21 (4.2) | 19 (4.9) |  | 10 (3.2) | 11 (3.5) |
| CT+TT | ]0, 37] | 14 (2.3) | 4 (1.3) |  | 89 (6.4) | 73 (5.7) |  | 8 (1.6) | 19 (4.9) |  | 10 (3.2) | 13 (4.1) |
| CT+TT | ]37, 45] | 24 (4.0) | 15 (5.0) |  | 82 (5.9) | 64 (5.0) |  | 18 (3.6) | 19 (4.9) |  | 23 (7.3) | 15 (4.8) |
| CT+TT | ]45, 53] | 20 (3.3) | 9 (3.0) |  | 81 (5.8) | 53 (4.1) |  | 8 (1.6) | 10 (2.6) |  | 12 (3.8) | 7 (2.2) |
| CT+TT | >53 | 26 (4.4) | 12 (4.0) |  | 46 (3.3) | 42 (3.3) |  | 29 (5.8) | 17 (4.4) |  | 16 (5.1) | 22 (7.0) |
|  |  |  |  |  |  |  |  |  |  |  |  |  |
