# Supplementary material for: Lack of Replication of the GRIN2A-by-Coffee Interaction in Parkinson Disease
Source: PLoS Genet. 2014 Nov 20;10(11):e1004788. doi: 10.1371/journal.pgen.1004788 (PMC4238979; doi:10.1371/journal.pgen.1004788)
Supplement: Table S5 — Independent and joint effects of coffee drinking and GRIN2A-rs4998386 for Parkinson disease by dataset. (DOCX) [file pgen.1004788.s005.docx]

Table S5. Independent and joint effects of coffee drinking and *GRIN2A*-rs4998386 for Parkinson’s disease by dataset.

|  |  | France | | | | | | | |  | | Denmark^a^ | | | | | | | | | |  | | Seattle-US | | | | | | |
| --- | --- | --- | --- | --- | --- | --- | --- | --- | --- | --- | --- | --- | --- | --- | --- | --- | --- | --- | --- | --- | --- | --- | --- | --- | --- | --- | --- | --- | --- | --- |
| \| *GRIN2A*- \| \| --- \| |  |  |  |  | | Interaction | |  | |  | |  | |  | | |  | Interaction | |  | |  | |  |  |  | | Interaction | |  |
| rs4998386 | Coffee | OR (95% CI) | p |  | | OR (95% CI) | | p | |  | | OR (95% CI) | | p | | |  | OR (95% CI) | | p | |  | | OR (95% CI) | p |  | | OR (95% CI) | | p |
| CC | Never | 1.00 (Ref.) | - |  | | - | | - | |  | | 1.00 (Ref.) | | - | | |  | - | | - | |  | | 1.00 (Ref.) | - |  | | - | | - |
| CC | Ever | 0.75 (0.51, 1.10) | 0.14 |  | | - | | - | |  | | 0.50 (0.33, 0.76) | | 0.001 | | |  | - | | - | |  | | 0.98 (0.70, 1.38) | 0.92 |  | | - | | - |
| CT, TT | Never | 1.25 (0.61, 2.54) | 0.54 |  | | 1.00 (Ref.) | | - | |  | | 3.88 (0.83, 18.22) | | 0.09 | | |  | 1.00 (Ref.) | | - | |  | | 1.10 (0.55, 2.19) | 0.80 |  | | 1.00 (Ref.) | | - |
| CT, TT | Ever | 0.79 (0.47, 1.31) | 0.36 |  | | 0.84 (0.37, 1.92) | | 0.68 | |  | | 0.42 (0.27, 0.66) | | <0.001 | | |  | 0.23 (0.02, 1.11) | | 0.08 | |  | | 1.46 (0.92, 2.31) | 0.11 |  | | 1.35 (0.61, 3.01) | | 0.46 |
|  |  |  |  |  | |  | |  | |  | |  | |  | | |  |  | |  | |  | |  |  |  | |  | |  |
|  | Cups per day | |  | |  | |  | |  | |  | |  | |  |  | | |  | |  | |  |  |  | |  | |  |  |
| CC | Never | 1.00 (Ref.) | - |  | | - | | - | |  | | 1.00 (Ref.) | | - | | |  | - | | - | |  | | 1.00 (Ref.) | - |  | | - | | - |
| CC | 1 | 0.77 (0.49, 1.22) | 0.27 |  | | - | | - | |  | | 1.00 (Ref.)^a^ | | - | | |  |  | |  | |  | | 1.16 (0.77, 1.75) | 0.49 |  | | - | | - |
| CC | 2 | 0.85 (0.54, 1.34) | 0.48 |  | | - | | - | |  | | 0.58 (0.43, 0.80) | | <0.001 | | |  | - | | - | |  | | 0.90 (0.61, 1.35) | 0.62 |  | | - | | - |
| CC | ≥3 | 0.64 (0.41, 1.02) | 0.06 |  | | - | | - | |  | | 0.50 (0.38, 0.65) | | <0.001 | | |  | - | | - | |  | | 0.85 (0.52, 1.38) | 0.51 |  | | - | | - |
| CT, TT | Never | 1.25 (0.61, 2.55) | 0.54 |  | | 1.00 (Ref.) | | - | |  | | 0.89 (0.50, 1.57) | | 0.69 | | |  | 1.00 (Ref.) | | - | |  | | 1.09 (0.55, 2.18) | 0.80 |  | | 1.00 (Ref.) | | - |
| CT, TT | 1 | 0.63 (0.30, 1.34) | 0.23 |  | | 0.65 (0.24, 1.82) | | 0.42 | |  | | 0.89 (0.50, 1.57) | | 0.69 | | |  | 1.00 (Ref.) | | - | |  | | 2.26 (1.15, 4.42) | 0.018 |  | | 1.78 (0.68, 4.71) | | 0.24 |
| CT, TT | 2 | 0.75 (0.33, 1.70) | 0.49 |  | | 0.70 (0.24, 2.07) | | 0.52 | |  | | 0.50 (0.32, 0.80) | | 0.004 | | |  | 0.97 (0.48, 1.99) | | 0.94 | |  | | 1.18 (0.64, 2.17) | 0.59 |  | | 1.20 (0.48, 2.98) | | 0.70 |
| CT, TT | ≥3 | 0.98 (0.50, 1.93) | 0.95 |  | | 1.21 (0.46, 3.21) | | 0.70 | |  | | 0.42 (0.31, 0.59) | | <0.001 | | |  | 0.96 (0.52, 1.78) | | 0.90 | |  | | 1.02 (0.43, 2.42) | 0.96 |  | | 1.10 (0.36, 3.40) | | 0.87 |
|  |  |  |  |  | | Global test | | 0.58 | |  | |  | |  | | |  | Global test | | 0.99 | |  | |  |  |  | | Global test | | 0.67 |
|  | Number of years of coffee drinking | | | | |  | |  | |  | |  | |  | | |  |  | |  | |  | |  |  |  | |  | |  |
| CC | Never | 1.00 (Ref.) | - |  | | - | | - | |  | | 1.00 (Ref.) | | - | | |  | - | | - | |  | | 1.00 (Ref.) | - |  | | - | | - |
| CC | ]0, 37] | 0.67 (0.39, 1.15) | 0.15 |  | | - | | - | |  | | 1.00 (Ref.)^a^ | | - | | |  |  | |  | |  | | 1.17 (0.69, 1.99) | 0.55 |  | | - | | - |
| CC | ]37, 45] | 0.59 (0.35, 0.99) | 0.05 |  | | - | | - | |  | | 0.83 (0.66, 1.05) | | 0.12 | | |  | - | | - | |  | | 0.87 (0.53, 1.44) | 0.59 |  | | - | | - |
| CC | ]45, 53] | 0.86 (0.54, 1.37) | 0.53 |  | | - | | - | |  | | 0.82 (0.65, 1.05) | | 0.12 | | |  | - | | - | |  | | 1.00 (0.56, 1.78) | 0.99 |  | | - | | - |
| CC | >53 | 0.84 (0.51, 1.37) | 0.48 |  | | - | | - | |  | | 0.69 (0.51, 0.93) | | 0.01 | | |  | - | | - | |  | | 0.92 (0.57, 1.47) | 0.72 |  | | - | | - |
| CT, TT | Never | 1.25 (0.61, 2.55) | 0.54 |  | | 1.00 (Ref.) | | - | |  | | 0.85 (0.61, 1.18) | | 0.34 | | |  | 1.00 (Ref.) | | - | |  | | 1.09 (0.55, 2.19) | 0.80 |  | | - | | - |
| CT, TT | ]0, 37] | 0.45 (0.14, 1.44) | 0.18 |  | | 0.54 (0.13, 2.14) | | 0.38 | |  | | 0.85 (0.61, 1.18) | | 0.34 | | |  | 1.00 (Ref.) | | - | |  | | 2.85 (1.13, 7.20) | 0.026 |  | | 2.22 (0.71, 6.96) | | 0.17 |
| CT, TT | ]37, 45] | 1.00 (0.48, 2.10) | 0.99 |  | | 1.35 (0.48, 3.81) | | 0.57 | |  | | 0.67 (0.47, 0.97) | | 0.03 | | |  | 0.95 (0.58, 1.57) | | 0.84 | |  | | 1.30 (0.62, 2.73) | 0.49 |  | | 1.37 (0.50, 3.76) | | 0.55 |
| CT, TT | ]45, 53] | 0.78 (0.33, 1.86) | 0.58 |  | | 0.73 (0.24, 2.20) | | 0.57 | |  | | 0.60 (0.41, 0.88) | | 0.01 | | |  | 0.85 (0.51, 1.42) | | 0.54 | |  | | 1.84 (0.67, 5.05) | 0.24 |  | | 1.68 (0.48, 5.88) | | 0.42 |
| CT, TT | >53 | 0.77 (0.36, 1.67) | 0.51 |  | | 0.74 (0.26, 2.10) | | 0.57 | |  | | 0.84 (0.53, 1.34) | | 0.46 | | |  | 1.43 (0.80, 2.54) | | 0.23 | |  | | 0.98 (0.48, 1.98) | 0.95 |  | | 0.97 (0.37, 2.56) | | 0.96 |
|  |  |  |  |  | | Global test | | 0.65 | |  | |  | |  | | |  | Global test | | 0.42 | |  | |  |  |  | | Global test | | 0.58 |

Table S5 (follows).

|  |  | France | | | | |  | Denmark^a^ | | | | |  |  | Seattle-US | | | | |
| --- | --- | --- | --- | --- | --- | --- | --- | --- | --- | --- | --- | --- | --- | --- | --- | --- | --- | --- | --- |
| \| *GRIN2A*- \| \| --- \| |  |  |  |  | Interaction |  |  |  |  |  | Interaction |  |  |  |  |  |  | Interaction |  |
| rs4998386 | Coffee | OR (95% CI) | p |  | OR (95% CI) | p |  | OR (95% CI) | p |  | OR (95% CI) | p |  |  | OR (95% CI) | p |  | OR (95% CI) | p |
|  | Cupyears |  |  |  |  |  |  |  |  |  |  |  |  |  |  |  |  |  |  |
| CC | Never | 1.00 (Ref.) | - |  | - | - |  | 1.00 (Ref.) | - |  | - | - |  |  | 1.00 (Ref.) | - |  | - | - |
| CC | ]0, 65] | 0.67 (0.43, 1.06) | 0.09 |  | - | - |  | 1.00 (Ref.)^a^ | - |  |  |  |  |  | 1.2 (0.8, 1.81) | 0.38 |  | - | - |
| CC | ]65, 130] | 0.96 (0.61, 1.51) | 0.87 |  | - | - |  | 0.76 (0.59, 1.00) | 0.05 |  | - | - |  |  | 0.93 (0.55, 1.58) | 0.78 |  | - | - |
| CC | ]130, 200] | 0.65 (0.39, 1.08) | 0.10 |  | - | - |  | 0.68 (0.52, 0.90) | 0.01 |  | - | - |  |  | 0.86 (0.54, 1.37) | 0.53 |  | - | - |
| CC | >200 | 0.62 (0.30, 1.27) | 0.19 |  | - | - |  | 0.57 (0.44, 0.74) | <0.001 |  | - | - |  |  | 0.77 (0.46, 1.29) | 0.31 |  | - | - |
| CT, TT | Never | 1.25 (0.61, 2.54) | 0.54 |  | 1.00 (Ref.) | - |  | 1.04 (0.65, 1.66) | 0.88 |  | 1.00 (Ref.) | - |  |  | 1.10 (0.55, 2.19) | 0.80 |  | 1.00 (Ref.) | - |
| CT, TT | ]0, 65] | 0.61 (0.28, 1.32) | 0.21 |  | 0.73 (0.26, 2.06) | 0.55 |  | 1.04 (0.65, 1.66) | 0.88 |  | 1.00 (Ref.) | - |  |  | 2.18 (1.13, 4.22) | 0.020 |  | 1.66 (0.64, 4.32) | 0.30 |
| CT, TT | ]65, 130] | 0.93 (0.44, 1.96) | 0.85 |  | 0.78 (0.28, 2.14) | 0.62 |  | 0.55 (0.37, 0.83) | 0.01 |  | 0.70 (0.38, 1.29) | 0.25 |  |  | 1.52 (0.67, 3.45) | 0.32 |  | 1.50 (0.49, 4.53) | 0.48 |
| CT, TT | ]130, 200] | 0.54 (0.23, 1.30) | 0.17 |  | 0.67 (0.22, 2.08) | 0.49 |  | 0.45 (0.30, 0.68) | <0.001 |  | 0.64 (0.35, 1.18) | 0.15 |  |  | 0.91 (0.39, 2.12) | 0.83 |  | 0.97 (0.32, 2.88) | 0.95 |
| CT, TT | >200 | 2.37 (0.71, 7.91) | 0.16 |  | 3.09 (0.69, 13.82) | 0.14 |  | 0.61 (0.43, 0.87) | 0.01 |  | 1.03 (0.58, 1.81) | 0.93 |  |  | 0.98 (0.40, 2.41) | 0.96 |  | 1.17 (0.36, 3.75) | 0.80 |
|  |  |  |  |  | Global test | 0.35 |  |  |  |  | Global test | 0.19 |  |  |  |  |  | Global test | 0.80 |
|  |  |  |  |  |  |  |  |  |  |  |  |  |  |  |  |  |  |  |  |
| CC | Light | 1.00 (Ref.) | - |  | - | - |  | 1.00 (Ref.) | - |  | - | - |  |  | 1.00 (Ref.) | - |  | - | - |
| CC | Heavy | 0.91 (0.66, 1.26) | 0.59 |  | - | - |  | 0.71 (0.60, 0.85) | <0.001 |  | - | - |  |  | 0.78 (0.55, 1.09) | 0.14 |  | - | - |
| CT, TT | Light | 1.12 (0.70, 1.78) | 0.63 |  | 1.00 (Ref.) | - |  | 0.75 (0.58, 0.97) | 0.03 |  | 1.00 (Ref.) | - |  |  | 1.49 (0.99, 2.25) | 0.058 |  | 1.00 (Ref.) | - |
| CT, TT | Heavy | 1.02 (0.59, 1.75) | 0.95 |  | 0.99 (0.48, 2.06) | 0.99 |  | 0.72 (0.55, 0.96) | 0.02 |  | 1.36 (0.93, 2.00) | 0.12 |  |  | 0.88 (0.48, 1.63) | 0.69 |  | 0.76 (0.36, 1.62) | 0.48 |
|  |  |  |  |  |  |  |  |  |  |  |  |  |  |  |  |  |  |  |  |
|  |  |  |  |  |  |  |  |  |  |  |  |  |  |  |  |  |  |  |  |
| CC | [0%, 25%] | 1.00 (Ref.) | - |  | - | - |  | 1.00 (Ref.) | - |  | - | - |  |  | 1.00 (Ref.) | - |  | - | - |
| CC | ]25%, 50%] | 0.77 (0.49, 1.19) | 0.24 |  | - | - |  | 0.86 (0.68, 1.08) | 0.20 |  | - | - |  |  | 1.15 (0.77, 1.71) | 0.49 |  | - | - |
| CC | ]50%, 75%] | 1.03 (0.68, 1.56) | 0.90 |  | - | - |  | 0.69 (0.54, 0.88) | 0.002 |  | - | - |  |  | 0.85 (0.55, 1.30) | 0.44 |  | - | - |
| CC | ]75%, 100%] | 0.63 (0.40, 0.99) | 0.05 |  | - | - |  | 0.60 (0.47, 0.77) | <0.001 |  | - | - |  |  | 0.88 (0.54, 1.41) | 0.58 |  | - | - |
| CT, TT | [0%, 25%] | 0.99 (0.53, 1.86) | 0.98 |  | 1.00 (Ref.) | - |  | 0.97 (0.68, 1.39) | 0.87 |  | 1.00 (Ref.) | - |  |  | 1.09 (0.55, 2.18) | 0.80 |  | 1.00 (Ref.) | - |
| CT, TT | ]25%, 50%] | 0.77 (0.35, 1.72) | 0.53 |  | 1.01 (0.36, 2.83) | 0.98 |  | 0.46 (0.32, 0.67) | <0.001 |  | 0.56 (0.33, 0.94) | 0.03 |  |  | 2.10 (1.13, 3.92) | 0.020 |  | 1.67 (0.66, 4.23) | 0.28 |
| CT, TT | ]50%, 75%] | 1.00 (0.47, 2.10) | 0.99 |  | 0.98 (0.37, 2.60) | 0.97 |  | 0.66 (0.46, 0.96) | 0.03 |  | 1.00 (0.59, 1.67) | 0.99 |  |  | 1.10 (0.57, 2.13) | 0.80 |  | 1.19 (0.46, 3.11) | 0.72 |
| CT, TT | ]75%, 100%] | 1.03 (0.51, 2.08) | 0.95 |  | 1.65 (0.63, 4.32) | 0.31 |  | 0.67 (0.44, 1.01) | 0.06 |  | 1.14 (0.65, 1.98) | 0.65 |  |  | 1.08 (0.47, 2.48) | 0.86 |  | 1.13 (0.38, 3.39) | 0.83 |
|  |  |  |  |  | Global test | 0.71 |  |  |  |  | Global test | 0.050 |  |  |  |  |  | Global test | 0.72 |

Odds ratios (OR) and 95% confidence intervals computed using unconditional logistic regression and adjusted for sex, age in quartiles, and ever cigarette smoking. Results for the Rochester-US dataset are shown in table 2.

^a^ We combined never drinkers with participants in the first category of exposure, due to the very small number of never drinkers who carried the T allele (2 controls, 14 cases; Table S4).
