# Supplementary material for: Lack of Replication of the GRIN2A-by-Coffee Interaction in Parkinson Disease
Source: PLoS Genet. 2014 Nov 20;10(11):e1004788. doi: 10.1371/journal.pgen.1004788 (PMC4238979; doi:10.1371/journal.pgen.1004788)
Supplement: Table S6 — Pooled analysis of independent and joint effects of coffee drinking and GRIN2A-rs4998386 for Parkinson disease: alternative definitions of coffee drinking. (DOCX) [file pgen.1004788.s006.docx]

Table S6. Pooled analysis of independent and joint effects of coffee drinking and *GRIN2A*-rs4998386 for Parkinson’s disease: alternative definitions of coffee drinking.

|  |  | France, Denmark, Seattle-US | | | | |  | Rochester-US | | | | |  | Pooled analysis | | | | |
| --- | --- | --- | --- | --- | --- | --- | --- | --- | --- | --- | --- | --- | --- | --- | --- | --- | --- | --- |
| \| *GRIN2A*- \| \| --- \| |  |  |  |  | Interaction | |  |  |  |  | Interaction | |  |  |  |  | Interaction | |
| rs4998386 | Coffee | OR (95% CI)^a^ | p |  | OR (95% CI)^a^ | p |  | OR (95% CI)^b^ | p |  | OR (95% CI)^b^ | p |  | OR (95% CI)^c^ | p |  | OR (95% CI)^c^ | p |
|  | Cupyears | |  |  |  |  |  |  |  |  |  |  |  |  |  |  |  |  |
| CC | Light | 1.00 (Ref.) | - |  | - | - |  | 1.00 (Ref.) | - |  | - | - |  | 1.00 (Ref.) | - |  | - | - |
| CC | Heavy | 0.76 (0.66, 0.87) | <0.001 |  | - | - |  | 1.11 (0.73, 1.69) | 0.63 |  | - | - |  | 0.77 (0.68, 0.88) | <0.001 |  | - | - |
| CT, TT | Light | 0.94 (0.78, 1.15) | 0.56 |  | 1.00 (Ref.) | - |  | 0.73 (0.38, 1.38) | 0.33 |  | 1.00 (Ref.) | - |  | 0.91 (0.76, 1.10) | 0.34 |  | 1.00 (Ref.) | - |
| CT, TT | Heavy | 0.80 (0.60, 3.10) | 0.053 |  | 1.11 (0.82, 1.51) | 0.50 |  | 1.34 (0.62, 2.86) | 0.45 |  | 1.66 (0.73, 3.76) | 0.23 |  | 0.85 (0.68, 1.05) | 0.13 |  | 1.19 (0.90, 1.59) | 0.22 |
| CT, TT | Heavy |  | EBI^d^ |  | 1.11 (0.88, 1.40) | 0.37 |  |  |  |  |  |  |  |  |  |  |  |  |
|  |  |  |  |  |  |  |  |  |  |  |  |  |  |  |  |  |  |  |
| CC | [0%,25%] | 1.00 (Ref.) | - |  | - | - |  | 1.00 (Ref.) |  |  | - | - |  | 1.00 (Ref.) |  |  | - | - |
| CC | ]25%, 50%] | 0.90 (0.75, 1.07) | 0.24 |  | - | - |  | 0.93 (0.53, 1.61) | 0.79 |  | - | - |  | 0.88 (0.74, 1.05) | 0.15 |  | - | - |
| CC | ]50%,75%] | 0.77 (0.64, 0.93) | 0.007 |  | - | - |  | 1.21 (0.69, 2.12) | 0.51 |  | - | - |  | 0.80 (0.67, 0.95) | 0.013 |  | - | - |
| CC | ]75%,100%] | 0.65 (0.54, 0.80) | <0.001 |  | - | - |  | 1.11 (0.62, 1.99) | 0.73 |  | - | - |  | 0.68 (0.56, 0.81) | <0.001 |  | - | - |
| CT, TT | [0%,25%] | 1.00 (0.76, 1.33) | 0.98 |  | 1.00 (Ref.) | - |  | 1.39 (0.57, 3.39) | 0.47 |  | 1.00 (Ref.) | - |  | 1.05 (0.81, 1.37) | 0.72 |  | 1.00 (Ref.) | - |
| CT, TT | ]25%, 50%] | 0.71 (0.53, 0.95) | 0.023 |  | 0.79 (0.53, 1.19) | 0.26 |  | 0.15 (0.04, 0.57) | 0.01 |  | 0.11 (0.02, 0.53) | 0.006 |  | 0.64 (0.48, 0.84) | 0.0016 |  | 0.69 (0.47, 1.01) | 0.056 |
| CT, TT | ]50%,75%] | 0.78 (0.58, 1.05) | 0.11 |  | 1.01 (0.67, 1.53) | 0.96 |  | 0.98 (0.38, 2.49) | 0.96 |  | 0.58 (0.18, 1.91) | 0.37 |  | 0.80 (0.60, 1.05) | 0.11 |  | 0.95 (0.65, 1.40) | 0.79 |
| CT, TT | ]75%,100%] | 0.80 (0.57, 1.11) | 0.18 |  | 1.21 (0.78, 1.89) | 0.39 |  | 1.47 (0.55, 3.92) | 0.44 |  | 0.95 (0.31, 2.97) | 0.93 |  | 0.87 (0.64, 1.18) | 0.36 |  | 1.22 (0.81, 1.83) | 0.34 |
|  |  |  |  |  | Global test^e^ | 0.31 |  |  |  |  | Global test^e^ | 0.01 |  |  |  |  | Global test^e^ | 0.055 |
|  |  |  |  |  |  |  |  |  |  |  |  |  |  |  |  |  |  |  |
| CT, TT | ]25%, 50%] |  | EBI^d^ |  | 0.87 (0.60, 1.28) | 0.49 |  |  |  |  |  |  |  |  |  |  |  |  |
| CT, TT | ]50%,75%] |  | EBI^d^ |  | 1.10 (0.79, 1.55) | 0.57 |  |  |  |  |  |  |  |  |  |  |  |  |
| CT, TT | ]75%,100%] |  | EBI^d^ |  | 1.10 (0.75, 1.60) | 0.62 |  |  |  |  |  |  |  |  |  |  |  |  |
|  |  |  | EBI^d^ |  | Global test^e^ | 0.61 |  |  |  |  |  |  |  |  |  |  |  |  |

^a^ Odds ratios (OR) and 95% confidence intervals computed using unconditional logistic regression and adjusted for sex, age in quartiles, ever cigarette smoking, and dataset.

^b^ Odds ratios (OR) and 95% confidence intervals computed using conditional logistic regression and adjusted for sex, age in quartiles, and ever cigarette smoking.

^c^ Odds ratios (OR) and 95% confidence intervals computed by pooling individual data from the matched and unmatched case-control analyses and adjusted for sex, age in quartiles, ever cigarette smoking, and dataset.

^d^ EBI, empirical Bayes interaction: odds ratios (OR) and 95% confidence intervals computed using an empirical Bayes approach.

^e^ Global test of interaction.
