# Supplementary material for: Lack of Replication of the GRIN2A-by-Coffee Interaction in Parkinson Disease
Source: PLoS Genet. 2014 Nov 20;10(11):e1004788. doi: 10.1371/journal.pgen.1004788 (PMC4238979; doi:10.1371/journal.pgen.1004788)
Supplement: Table S7 — Pooled analysis of independent and joint effects of coffee drinking and GRIN2A-rs4998386 for Parkinson disease: empirical Bayes approach for estimating interactions. (DOCX) [file pgen.1004788.s007.docx]

Table S7. Pooled analysis of independent and joint effects of coffee drinking and *GRIN2A*-rs4998386 for Parkinson’s disease: empirical Bayes approach for estimating interactions.

|  |  | France, Denmark, Seattle-US | | | | |
| --- | --- | --- | --- | --- | --- | --- |
| *GRIN2A*- |  |  |  |  | EB Interaction | |
| rs4998386 | Coffee | OR (95% CI)^a^ | p |  | OR (95% CI)^b^ | p |
|  | Ever vs never | |  |  |  |  |
| CC | Never | 1.00 (Ref.) | - |  | - | - |
| CC | Ever | 0.77 (0.62, 0.96) | 0.017 |  | - | - |
| CT, TT | Never | 1.32 (0.84, 2.07) | 0.23 |  | 1.00 (Ref.) | - |
| CT, TT | Ever | 0.73 (0.57, 0.94) | 0.014 |  | 0.81 (0.49, 1.34) | 0.41 |
|  |  |  |  |  |  |  |
|  | Cups per day | |  |  |  |  |
| CC | Never | 1.00 (Ref.) | - |  | - | - |
| CC | 1 cup | 0.98 (0.76, 1.26) | 0.87 |  | - | - |
| CC | 2 cups | 0.77 (0.61, 0.98) | 0.034 |  | - | - |
| CC | ≥3 cups | 0.64 (0.51, 0.81) | <0.001 |  | - | - |
| CT, TT | Never | 1.33 (0.85, 2.09) | 0.22 |  | 1.00 (Ref.) | - |
| CT, TT | 1 cup | 0.99 (0.67, 1.47) | 0.96 |  | 0.89 (0.49, 1.59) | 0.69 |
| CT, TT | 2 cups | 0.75 (0.53, 1.07) | 0.11 |  | 0.84 (0.49, 1.45) | 0.54 |
| CT, TT | ≥3 cups | 0.59 (0.44, 0.79) | <0.001 |  | 0.78 (0.46, 1.32) | 0.34 |
|  |  |  |  |  | Global test^c^ | 0.81 |
|  |  | |  |  |  |  |
|  | Cupyears | |  |  |  |  |
| CC | Never | 1.00 (Ref.) | - |  | - | - |
| CC | ]0, 65] | 0.88 (0.69, 1.12) | 0.30 |  | - | - |
| CC | ]65, 130] | 0.85 (0.67, 1.09) | 0.20 |  | - | - |
| CC | ]130, 200] | 0.70 (0.55, 0.90) | 0.006 |  | - | - |
| CC | >200 | 0.61 (0.48, 0.79) | <0.001 |  | - | - |
| CT, TT | Never | 1.33 (0.85, 2.08) | 0.22 |  | 1.00 (Ref.) | - |
| CT, TT | ]0, 65] | 0.98 (0.68, 1.42) | 0.93 |  | 0.97 (0.58, 1.62) | 0.91 |
| CT, TT | ]65, 130] | 0.72 (0.51, 1.03) | 0.073 |  | 0.74 (0.42, 1.31) | 0.30 |
| CT, TT | ]130, 200] | 0.52 (0.36, 0.74) | <0.001 |  | 0.63 (0.35, 1.13) | 0.12 |
| CT, TT | >200 | 0.71 (0.51, 1.00) | 0.049 |  | 1.01 (0.59, 1.72) | 0.99 |
|  |  |  |  |  | Global test^c^ | 0.24 |
|  |  | | |  |  |  |
|  | Number of years of coffee drinking | | |  |  |  |
| CC | Never | 1.00 (Ref.) | - |  | - | - |
| CC | ]0, 37] | 0.84 (0.65, 1.08) | 0.17 |  | - | - |
| CC | ]37, 45] | 0.74 (0.58, 0.95) | 0.018 |  | - | - |
| CC | ]45, 53] | 0.80 (0.63, 1.03) | 0.084 |  | - | - |
| CC | >53 | 0.71 (0.55 ,0.92) | 0.011 |  | - | - |
| CT, TT | Never | 1.32 (0.84, 2.06) | 0.23 |  | 1.00 (Ref.) | - |
| CT, TT | ]0, 37] | 0.76 (0.53, 1.08) | 0.12 |  | 0.79 (0.46, 1.35) | 0.39 |
| CT, TT | ]37, 45] | 0.75 (0.53, 1.05) | 0.093 |  | 0.87 (0.49, 1.55) | 0.64 |
| CT, TT | ]45, 53] | 0.66 (0.46, 0.95) | 0.025 |  | 0.72 (0.40, 1.28) | 0.26 |
| CT, TT | >53 | 0.79 (0.55, 1.14) | 0.21 |  | 0.97 (0.57, 1.63) | 0.90 |
|  |  |  |  |  | Global test^c^ | 0.72 |

^a^ Odds ratios (OR) and 95% confidence intervals computed using unconditional logistic regression and adjusted for sex, age in quartiles, ever cigarette smoking, and dataset.

^b^ Interaction odds ratios (OR) and 95% confidence intervals computed using an empirical Bayes (EB) approach.

^c^ Global test of interaction.
